# TL-iGeneCombo: transfer learning for individual sample specific gene combination effect prediction and selection in cancer cells

**DOI:** 10.1093/bioinformatics/btag760

**Published:** 2026-10-10

**Authors:** Birkan Gökbağ, Lingling Wang, Chen Yang, Yun Shen, Ping Zhang, Lijun Cheng, Lang Li

**Affiliations:** Department of Biomedical Informatics, College of Medicine, The Ohio State University, Columbus, OH 43210, United States; Department of Biomedical Informatics, College of Medicine, The Ohio State University, Columbus, OH 43210, United States; Department of Biomedical Informatics, College of Medicine, The Ohio State University, Columbus, OH 43210, United States; Department of Biomedical Informatics, College of Medicine, The Ohio State University, Columbus, OH 43210, United States; Department of Biomedical Informatics, College of Medicine, The Ohio State University, Columbus, OH 43210, United States; Department of Biomedical Informatics, College of Medicine, The Ohio State University, Columbus, OH 43210, United States; Department of Biomedical Informatics, College of Medicine, The Ohio State University, Columbus, OH 43210, United States

## Abstract

**Motivation:**

Identifying effective gene combinations is critical for developing combinatorial cancer therapies. Synthetic lethality (SL) has been widely used to characterize genetic interactions based on cell viability, but predictive models are often limited by the availability of combinatorial double-knockout (CDKO) data for individual cancer cells. How to borrow information from CDKO experiments in other cancer cells to improve prediction in a targeted cancer cell remains an important challenge.

**Results:**

We investigated the CDKO effects of paired genes using both SL as an indirect measure and gene combination effect as a direct measure. We developed TL-iGeneCombo, a transfer learning approach, to borrow data from CDKO experiments in other cancer cells for the prediction of SL and gene combination effect in a targeted cancer cell. Applying TL-iGeneCombo improved the accuracy of SL classification by 19% and increased the Spearman correlation of gene combination effect scores by 29%. Using data and models built from the first CDKO experiment in a prostate cancer cell, 22RV1, we further designed gene pairs for a second CDKO experiment in the same cell. It validated the superior prediction of gene combination effect, but not SL prediction.

**Availability and implementation:**

The software is freely available at https://github.com/BirkanGokbag/TL-iGeneCombo under the GNU General Public License v.3.0.

## Graphical abstract

**Graphical Abstract:**
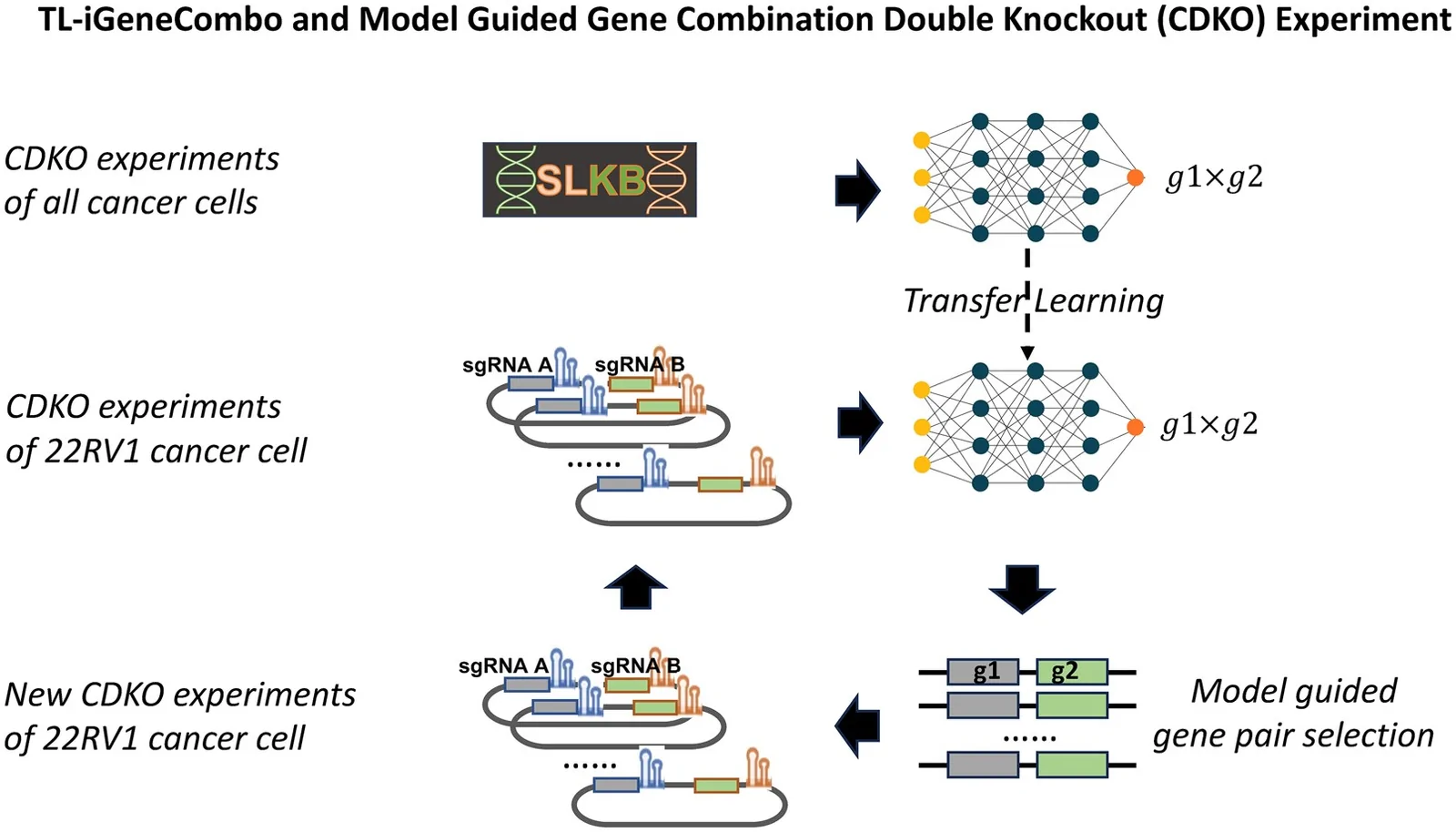

## 1 Introduction

Cancer is a complex disease in which multiple mechanisms can coexist, and this heterogeneity motivates researchers to investigate the most effective gene combinations for combinatorial therapies in specific cancers (Dagogo-Jack and Shaw 2018). In the literature of computational biology and systems biology, gene combination discovery relies on how the gene-combinatorial effect is defined. One approach to identify such combinational targets utilizes network models, analyzing network graphs. Examples include virtual inference of protein activity by enriched regulon analysis (VIPER) (Paull *et al.* 2021), which examines the signaling pathway network, the identification of optimal control nodes (OptiCon) (Hu *et al.* 2019) within the gene regulatory network, and double-target selection guided by CRISPR screening and network (DSCN) (Liu *et al.* 2022a), which scrutinizes the protein–protein interaction network. The utility of these methods in uncovering effective gene combinations is reflected in their impact on the biological network. In these network models, gene combination discovery is carried out at the population level, not for an individual sample, and the selection of gene combinations is not based on cell viability.

Synthetic lethality (SL), on the other hand, defines the effect of gene combination according to cell viability. SL depicts the relationship between paired genes in which perturbation of both genes, not just one, causes the host cell to die (O’Neil et al. 2017). Several approaches have been developed for the prediction of SL. One important approach, statistical inference, relies on biological assumptions of SL gene pairs and calculates the probability of SL using multiomics data and single-gene siRNA or CRISPR-Cas9 screening data. Examples include the data mining SL identification pipeline (DAISY) (Jerby-Arnon *et al.* 2014), mining synthetic lethals algorithm (Sinha *et al.* 2017), and identification of clinically relevant SL (Lee *et al.* 2018). Another type of SL prediction model is based on machine learning (ML) and artificial intelligence (AI) methods. Unlike the statistical inference approach, these methods are trained in a supervised manner, relying on known SL and non-SL data, such as SynLethDB (Wang *et al.* 2022). Important representative methods include Mashup (Cho *et al.* 2016), collective matrix factorization (Liany *et al.* 2020), dynamic directed graph convolutional network (Cai *et al.* 2020), graph contextualized attention network for the prediction of SL (Long *et al.* 2021), and knowledge graph for SL (Zhang *et al.* 2023) as well as the pairwise interaction learning-based graph neural network model for SL (Liu *et al.* 2022b). Limited by the training data in the SynLethDB, these ML/AI methods can predict SL only at the population level and not for an individual sample.

Recently, we developed a new class of models purposely designed for the prediction of SL in individual samples (iSL), including a multi-view graph convolutional network (MVGCN-iSL) (Fan *et al.* 2023) and multilayer encoders (MLEC-iSL) (Fan *et al.* 2021). The addition of a transformer layer into the MVGCN-iSL model considerably improved SL prediction with MLEC-iSL in two cancer cell lines, K562 and Jurkat, as reflected in the area under the receiver-operating characteristic curve (AUC-ROC) scores between MVGCN-iSL (0.62) and MLEC-iSL (0.72). Unlike population-based SL prediction models, these individual SL prediction models use sample-specific multiomics data as input features. The rich data generated from CRISPR-Cas9 CDKO (combinatorial double-knockout) experiments made these models feasible, and the data of all these published CDKO experiments were recently integrated in the new Synthetic Lethality Knowledge Base (SLKB) (Gokbag *et al.* 2024).

Despite these early successes of SL prediction models for individual cancer cells, significant challenges and questions remain as to how these predictive models should be further developed and implemented in the selection of gene combinations. For instance, if a cancer cell has insufficient SL data to be trained in a machine-learning model, how can we borrow SL data and predictive models from other cancer cells? Is there an alternative to SL as a measure of gene combination effect that can be predicted from the multiomics data of individual cells? How does gene selection in a CDKO experiment lead to a better prediction model for gene combination effect?

To address these challenges, we proposed three key innovations. We first developed a new framework for SL prediction that utilized transfer learning (TL) to borrow data and models from other cancer cells. In computational biology research, the use of TL has been shown to improve the prediction of anticancer drug synergy in cell lines, with improvements of AUC-ROC scores of around 2 to 3% and of area under the precision-recall curve (AUPR) values of 5 to 9% (Kim *et al.* 2021). We then investigated whether SL prediction models could improve the accuracy of SL selection in a new CDKO experiment and whether TL could further improve prediction accuracy. To our knowledge, this is the first study in which SL gene pairs have been selected from a computational model in a CDKO experiment. In a CDKO experiment, dual-sgRNA sequence reads represent the direct measurement of cell viability before and after transfection of cancer cells with lentivirus containing a CRISPR-cas9 dual-sgRNA library. Finally, we explored the choice of log fold-change of a dual-sgRNA sequence read as the effect from the gene combination knockout. This provides a direct measurement of the effect of gene combinatory effect (GCE) on cell viability.

## 2 Materials and methods

### 2.1 TL scenarios in SL and GCE prediction

We considered four different TL scenarios (Fig. 1, available as supplementary data at *Bioinformatics* online). For each TL scenario, a source model was trained on the source data and then used in the target data and to train the TL model. A TL model was designed to investigate whether SL or GCE data from the source data can improve SL or GCE prediction in the target data. The application of TL in the design of a CDKO experiment requires that some SL or GCE data is available for the target cell. TL borrows the model from the source data and potentially provides better gene combination selection for a CDKO experiment in the target cell. In this paper, we focus on applying TL within, but not between, SL and GCE.

**Figure 1:**
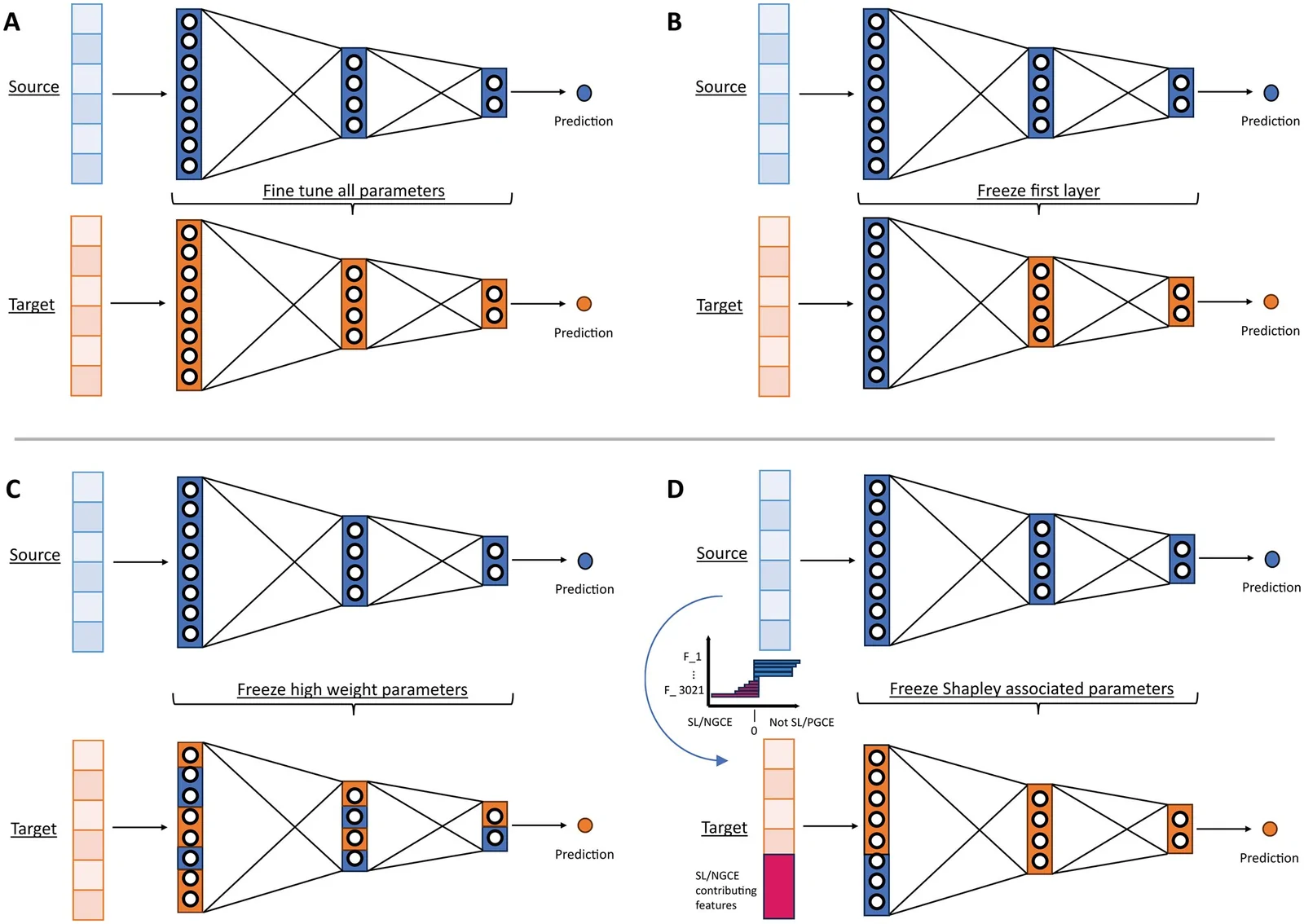
Summary of four TL methods. (a) Finetuning: all model parameters are retrained on the target data. (b) Freeze first layer: parameters in the first layer are frozen, and remaining parameters are retrained on the target data. (c) Freeze high weights: parameters close to 0 are retrained on the target data, and high-weight parameters are frozen. (d) Freeze Shapley: parameters associated with SL/gene combination effect are frozen, and remaining parameters are retrained in the target data. This applies only to the first layer, and the remaining layers are trained normally. (a) and (b) are existing TL methods, whereas (c) and (d) are designed for this study. NGCE, negative gene combination effect; PGCE, positive gene combination effect; SL, synthetic lethality.

The first application, same genes and different cell, applies TL from the source data to the target data that share the same gene pairs between two different cell lines (Fig. 1A, available as supplementary data at *Bioinformatics* online). The second application, same cell and different genes, applies TL from the source data to the target data that share the same cell line but different gene pairs (Fig. 1B, available as supplementary data at *Bioinformatics* online). The third application, similar mechanism, conducts TL among CDKO data sets that share similar mechanisms of gene or gene-pair selection, such as signaling pathways, drug targets, paralog gene families, and others (Fig. 1C, Table 1, available as supplementary data at *Bioinformatics* online). In this scenario, the source data and target data may or may not have the same genes or cell lines. In the fourth application, big data to small data, the source model is trained with the entire SLKB data set with the target data held out. This scenario allows the source model to leverage most available data, and as a result, the source data may have the same gene or cells as the target data (Fig. 1D, available as supplementary data at *Bioinformatics* online).

### 2.2 CDKO experiment data and training/testing/validation data preparation for SL and GCE prediction model development

In developing the SL/GCE prediction model, we considered data of CDKO experiments integrated into the SLKB (Gokbag *et al.* 2024). Selection of the proper CDKO data sets required that the (i) CDKO data set included a relatively large number of gene pairs for the testing and validation data split and that (ii) cell lines in the CDKO data set included multiomics data. Therefore, in the first case, the data of Najm’s 2018 study (Najm *et al.* 2018) was removed from SL prediction tasks because of its low number of SL pairs, and in the second case, the 2017 study of Diehl and colleagues (Diehl *et al.* 2021) was removed completely, and the 2021 report of Thompson’s team (Thompson *et al.* 2021) was removed partially because of the absence of multiomics data for RPE-1 and HEK293T cells. We thus retained with a total of nine sets of CDKO experiment data in this paper (Table 1, available as supplementary data at *Bioinformatics* online). To generate cell-specific features, we collected multiomics data, including gene expression, copy numbers, and gene essentiality scores, from Dependency Map (DepMap) (Tsherniak *et al.* 2017).

We sequentially conducted two CDKO screens on the 22RV1 cell. In the first CDKO experiment, we selected 50 genes in cell-death pathways and tested all 1225 gene combinations (Tang *et al.* 2022). From these, we selected SL gene pairs by consensus from seven different SL scores (Gokbag *et al.* 2024), concluding with 88 SL pairings and 1137 non-SL pairs. This CDKO data set was included in the SLKB. Then, in the second CDKO experiment, we tested 151 genes and their selected 987 gene combinations from the apoptosis, autosis, and mitotic cell-death pathways. Using the same seven SL scores and consensus as in the first CDKO experiment data analysis, we concluded with 462 SL gene combinations and 525 non-SL pairings. Both CDKO studies shared nine genes (*AKT3, JUN, MAP2K1, MAPK1, MAPK3, MAPK8, NRAS, PARP1, and PIK3CA*), and nine gene pairs, and we conducted both CDKO experiments in triplicate. In the first CDKO experiment, each gene had three sgRNAs, and in the second CDKO experiment, each gene had two sgRNAs.

Development of the SL and GCE prediction models involved the preparation of training data sets in two different settings in which either a gene or gene combination was left out. In the leave gene combinations out setting, gene combinations were randomly grouped into training (70%), testing (20%), and validation (10%) data sets, and there was some overlapping of genes among them. In the leave genes out setting, on the other hand, 20% of genes were randomly selected from the dataset, and those genes and their combinations were excluded from the training set and stored in the testing set. As a result, in both settings, model capability was evaluated to generalize to new gene pairs (leave gene combinations out) and new genes (leave genes out). For SL prediction tasks, only genes that demonstrated SL in at least one gene pair were eligible for selection, and 20% of those genes were randomly selected.

In a separate model evaluation analysis, SL or GCE prediction models were developed using SLKB database or the first round CDKO experiment on a 22RV1 cell, and they are validated using the second round CDKO experiment on a 22RV1.

The GCE data of each study’s cell line was scaled to [−1, 1] to allow the regression values to be comparable across different data sets. This normalization allowed predictive models to be more transferable across different cell lines in TL.

### 2.3 Signature-iSL: molecular signature-based SL prediction model for individual samples

Though MVGCN-iSL and MLEC-iSL, our previously developed SL prediction models for individual samples, showed good prediction performance, they were not properly designed for the implementation of TL. Their graph-convolution layers and transformer layers were difficult to interpret and translate from the source model to the target model in TL, and it was also difficult to evaluate feature contributions to SL prediction in both models. Therefore, we decided to explore a simplified prediction model, the Signature-iSL model, a multilayer perceptron (MLP) (Fig. 2, available as supplementary data at *Bioinformatics* online). Gene features were initially represented as a vector of zeros and ones based on their gene-set membership from the molecular signature database (MsigDB) (Liberzon *et al.* 2011) and XDeathDB (Gadepalli *et al.* 2021). A molecular signature is assigned a value of one if gene is present in a gene set and zero otherwise. We curated a total of 33 883 gene sets for 6181 genes and maintained the top 1007 features that exhibited the highest variance among samples as the most informative molecular signatures. To generate cell-specific features, we collected multiomics data, including gene expressions, copy numbers, and gene essentiality scores, from the cancer DepMap (Tsherniak *et al.* 2017). Multiplication of the gene pair vectors by the corresponding multiomics data yielded 3021 × 1 vector (Fig. 2A, available as supplementary data at *Bioinformatics* online). Figure 2B, available as supplementary data at *Bioinformatics* online, illustrates the generation of gene-pair features by summing the combined features of the two genes, and Fig. 2C, available as supplementary data at *Bioinformatics* online, shows them being fed into a deep-learning model comprising a four-layered feedforward neural network. Without loss of generalization, the fours layers of the neural network were defined as $\{I,\;H_{1},\;H_{2},\;O\},$ consisting of the input layer {I}, with a dimension of 3021; the first hidden layer {*H_1_*}, with a dimension of 256; the second hidden layer {*H_2_*}, with a dimension of 128, and the output layer {*O*}, with a dimension of 1. Between the layers, the leaky rectified linear unit activation function (LeakyReLU) (Xu *et al.* 2015) was used with a dropout rate of 20% (Srivastava *et al.* 2014). In the final layer’s output, sigmoid activation was used for SL classification, and no activation was used for GCE regression (Narayan 1997). Details regarding gradient calculations for each layer and the applied TL method are outlined in Methods Section 1, available as supplementary data at *Bioinformatics* online.

**Figure 2:**
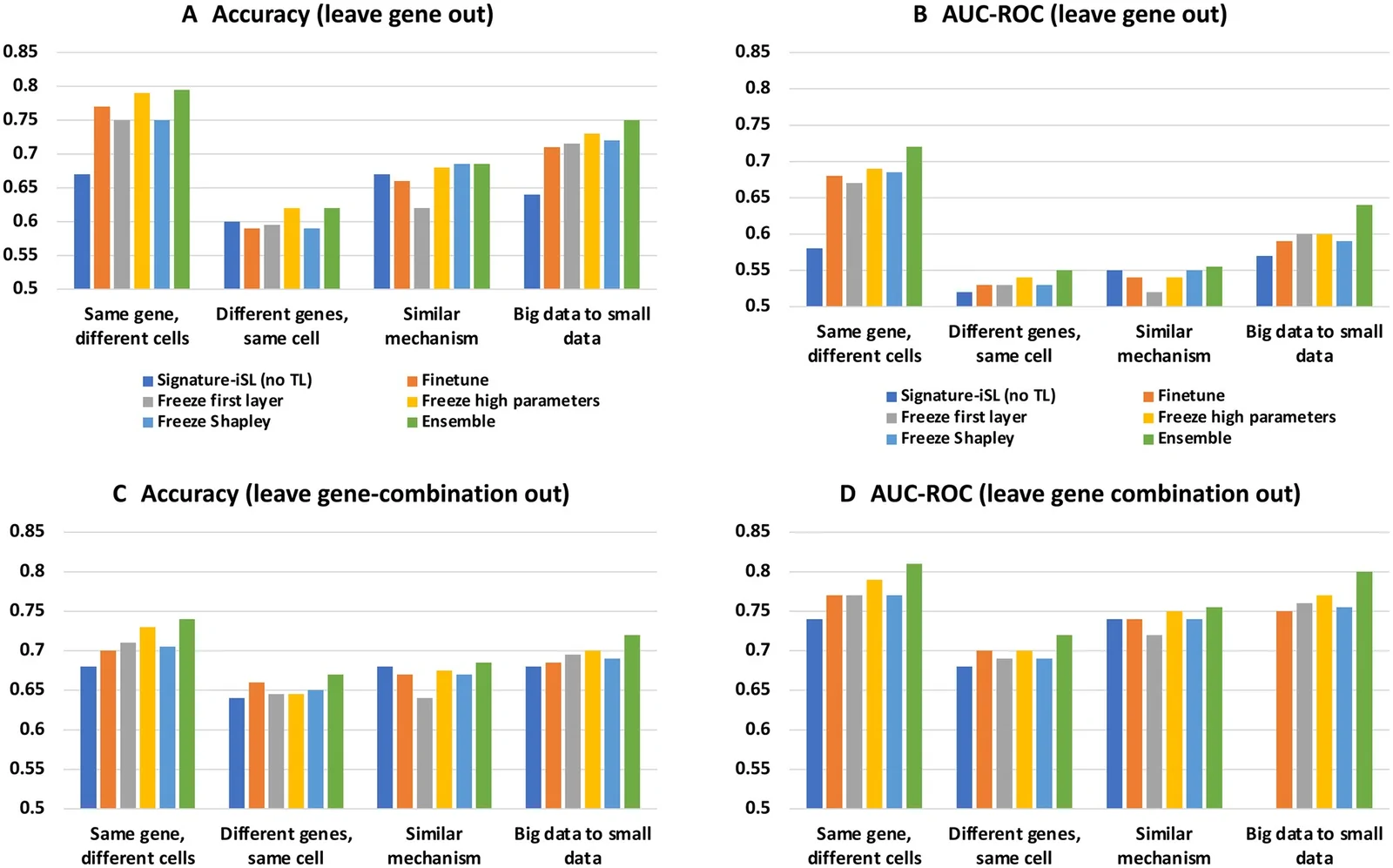
Performance comparison for SL prediction. Prediction performance is compared between the baseline model, Signature-iSL, and several TL methods, including finetune, freeze first layer, freeze high parameters, freeze Shapley, and the ensemble of the other four. Prediction performance is also compared among four TL scenarios, including same gene and different cells, same cell and different genes, similar mechanism, and big data to small data. (a) Accuracy for leave genes out; (b) AUC-ROC for leave genes out; (c) Accuracy for leave gene combinations out; and (d) AUC-ROC for leave gene combinations out.

### 2.4 Definitions of SL and GCE

SL prediction is a classification task, and GCE prediction is a regression task. For SL prediction, we utilized data of SL/non-SL gene pairs from studies included in the SLKB (Gokbag *et al.* 2024). No additional SL labels were generated in this study. We defined GCE using data sets of the CDKO experiments delineated in Table 2, available as supplementary data at *Bioinformatics* online. We filtered those dual-sgRNAs with fewer than 35 read counts in only the first time point ($T_{0}$) of a CDKO experiment and then updated the remaining dual-sgRNA counts with a pseudo count of 10. Afterwards, dual-sgRNA counts were normalized to the median of the total sum of counts across the $T_{0}$ and $T_{\mathrm{End}}$ timepoints among all replicated samples. GCE is defined as the median of the log_2_-fold-change (LFC) of dual-sgRNA sequence reads from a gene combination between $T_{\mathrm{End}}$ and $T_{0}$. Let J denote the number of experimental replicates. The LFC of a dual-sgRNA s and its targeting gene pair ${(g}_{1},g_{2})$ was calculated by:


(1) $${\mathrm{LFC}}_{g_{1}g_{2}s}=\log(\frac{{\mathrm{median}}_{j=1}^{J}\{Y_{g_{1}g_{2}sT_{\mathrm{end}}j}\}}{{\mathrm{median}}_{i=1}^{I}\{Y_{g_{1}g_{2}{sT}_{0}i}\}})$$

Then, the GCE of a gene combination was calculated by taking the median of its dual-sgRNA LFCs, and S is the total number of dual-sgRNAs for gene combination ${(g}_{1},g_{2})$:


(2) $${\mathrm{GCE}}_{g_{1}g_{2}}=\mathrm{media}n_{s=1}^{S}\{LFC_{g_{1}g_{2}s}\}$$

### 2.5 TL model speculation

In TL, $\left\{I_{S},{H_{1S},H_{2S},O}_{S},Y_{S}\right\}$ represent the input, two hidden layers, output (model prediction) and the observed data (i.e. SLs or GCEs); and $\left\{I_{T},H_{1T},H_{2T},{O_{T},Y}_{T}\right\}$ represent the corresponding outcome (labels). Between corresponding parameters in the source model and target model, i.e. $w_{S}=\{w_{SH_{1}},\;w_{SH_{2}},\;w_{SO}\}$ and $w_{T}=\{w_{TH_{1}},\;w_{TH_{2}},\;w_{TO}\}$, four TL strategies were designed. Each TL method shared two identical processes: (i) the initialization of target model parameters to be the same as those of the source model ($w_{T}=w_{S}$) prior to target model training and (ii) the use of the Adamax optimizer by each model to update layer parameters during backpropagation. The four constituent TL methods in the TL-iGeneCombo model that we implemented are outlined in Fig. 1 and below.

In finetuning, all parameters of the target model were retrained after the initial parameter values were set from the source model (Zhuang *et al.* 2021) (Fig. 1a). In freeze the first layer, parameters in the first layers of the target model were fixed to match those of the source model. See Equation (3) (Zhuang *et al.* 2021), in which $\hat{w}$ represents the estimate of w. Other parameters in the target model were trained in the target data (Fig. 1b).


(3) $${\hat{w}}_{TH_{1}}={\hat{w}}_{SH_{1}}$$

In freeze high parameters, parameters were fixed in the target model that demonstrated high absolute value of weight (impact) in the source model. Only parameters in the target model with low absolute value of weight in the source model were trained. By default, PyTorch uses a 32-bit floating point precision that causes the smallest gradients to be as low as $2^{-126}$. For every parameter *p* and loss function *L*, 0.01 is the threshold of the high weight. The gradients in the Jacobian matrix are updated with Equation (4) in the backpropagation algorithm (Fig. 1c).


(4) ∀l ∈{H1,H2,O}, ∀p ∈wl, dLdp={0 if p>|0.01|dLdp otherwise

Finally, in freeze Shapley, features were selected based on their contribution to prediction. The parameters directly impacted by SL or negative GCE skewing features were frozen, and the remaining parameters were retrained only in the first hidden layer (*H_1_*) (Fig. 1d). Because the first layer is a feedforward dense layer, features directly impact all rows of the layer’s parameter weights based on their location as a result of the layer’s matrix multiplicative step, for e.g., feature i directly impacts $w_{H1}i,j$ for $\forall j\in N^{256}$. Based on the game theory, Shapley values explain the impact of each input feature *I* in the final output *O* in machine-learning models (Sundararajan and Najmi 2020). Existing Shapley value methods such as DeepLIFT and G-DeepShap rely on imaging-style input formats and are not suitable for gene combination features in our SL and GCE prediction tasks (Shrikumar *et al.* 2017). Therefore, we developed a Shapley value algorithm to determine the impact of features on SL and GCE predictions (Methods Section 2, available as supplementary data at *Bioinformatics* online). The new Shapley value calculation was based on Monte Carlo sampling of present gene combination features.

For a series of Shapley values $\left\{S_{1},S_{2},\ldots S_{3021}\right\}$ that were calculated for each input feature $I\in \{I_{1},I_{2},\ldots I_{3021}\}$, SL features were frozen for SL prediction tasks, and negative GCE-related features were frozen for GCE prediction tasks. As a result, for every parameter *p* in the first hidden layer and loss function *L*, we disabled gradient calculation and thereby modified the Jacobian matrix by:


(5) dLdpij={0 if Si>0 in SL prediction, Si<0 in GCE predictiondLdpi,j otherwise∀p ∈wH1,∀i∈[1,…, 3021],∀j∈[1,…,256]

#### 2.5.1 Ensemble: ensemble prediction is the average among the four SL methods


(6) $$O_{\mathrm{ensemble}}=\frac{O_{\mathrm{finetune}}+O_{\mathrm{freeze}\_\mathrm{first}\_\mathrm{layer}}+O_{\mathrm{freeze}\_\mathrm{high}\_\mathrm{params}}+O_{\mathrm{freeze}\_\mathrm{shapley}}}{4}$$

#### 2.5.2 Source-data- and target-data-set speculation from the SLKB

Among all the published CDKO experiments, we selected data from nine studies for TL analysis (Table 1, available as supplementary data at *Bioinformatics* online). Because the 2017 study of Han and colleagues (Han *et al.* 2017) had no sgRNA counts to calculate GCE scores, the Han study was only available for SL prediction. Table 3, available as supplementary data at *Bioinformatics* online, outlines the total number of trained models for TL. We trained three types of models: (i) models trained without TL, which served as the benchmark for evaluating TL performance; (ii) models trained only for TL purposes and not used in TL evaluation, such as those trained using the entire data of the SLKB; and (iii) models trained using TL. In our experiments, we prepared each data set in two ways, in either a leave genes out split, in which at least one gene in the test gene pairs was not available in the training set, or a leave gene combinations out split, in which no such assumption was made. This allowed us to generalize leave genes out findings to out-of-sample genes and leave gene combinations out findings to predefined genes. Furthermore, we carried out each experiment five times with different seeds and randomly generated training, testing, and validation data sets in each experiment. As a result, training of each model was carried out ten times and for a total of 8,900 TL scenarios in SL prediction and 8,600 TL scenarios for GCE prediction. Methods Section 3, available as supplementary data at *Bioinformatics* online, presents more detailed source data and target data preparations in TL.

#### 2.5.3 SL and GCE prediction performance metrics

SL prediction performance was evaluated based on accuracy, precision, recall, AUC-ROC, AUPR, F1-score, AUC-ROC at 10%, 20%, and 50%, precision at the top 10%, 20%, and 50%, true positive rate, and false positive rate. We evaluated GCE prediction performance by both Spearman correlation and Pearson correlation.

## 3 Results

### 3.1 The baseline model, signature-iSL, showed better SL prediction than MLEC-iSL and MVGCN-iSL

Signature-iSL is a MLP deep-learning model for SL and GCE prediction, and it is our baseline model. Before we could integrate TL with Signature-iSL, we needed to compare its performance with that of MLEC-iSL (Fan *et al.* 2024) and MVGCN-iSL (Fan *et al.* 2023), our two existing SL prediction models for individual samples. These two SL prediction models employed features from both population-based and cell-specific multiomics profiles using graph convolutional networks (MVGCNs) and graph transformers (MLECs)**.** In our previous research, both the MLEC-iSL and MVGCN-iSL models achieved state-of-the-art SL prediction on the Jurkat and K562 cell lines from the CDKO experiment data reported by Horlbeck’s group in 2018 (Horlbeck *et al.* 2018), and the two models used similar test/validation data splits as those we implemented in this paper in their computational experiments, leaving out either genes or gene combinations.

Nonetheless, we evaluated the predictive performance of Signature-iSL against that of the MVGCN-iSL and MLEC-iSL approaches, conducting a total of 40 different experiment runs and using leave genes out and leave gene combinations out validation for the Jurkat and K562 cell lines**.** Signature-iSL outperformed both MVGCN-iSL and MLEC-iSL. In the leave genes out setting, Signature-iSL reached 0.75 scores for both AUC-ROC and AUPR compared to values for MVGCN-iSL (AUC-ROC, 0.62; AUPR, 0.63) and MLEC-iSL (AUC-ROC, 0.65; AUPR, 0.66). In the leave gene combinations out setting, Signature-iSL also outperformed the other two methods, with AUC-ROC and AUPR scores of 0.90 and 0.89 compared to the scores for MVGCN-iSL (AUC-ROC, 0.84; AUPR, 0.83) and MLEC (AUC-ROC, 0.82; AUPR, 0.77). These results are shown in Fig. 3, available as supplementary data at *Bioinformatics* online.

**Figure 3:**
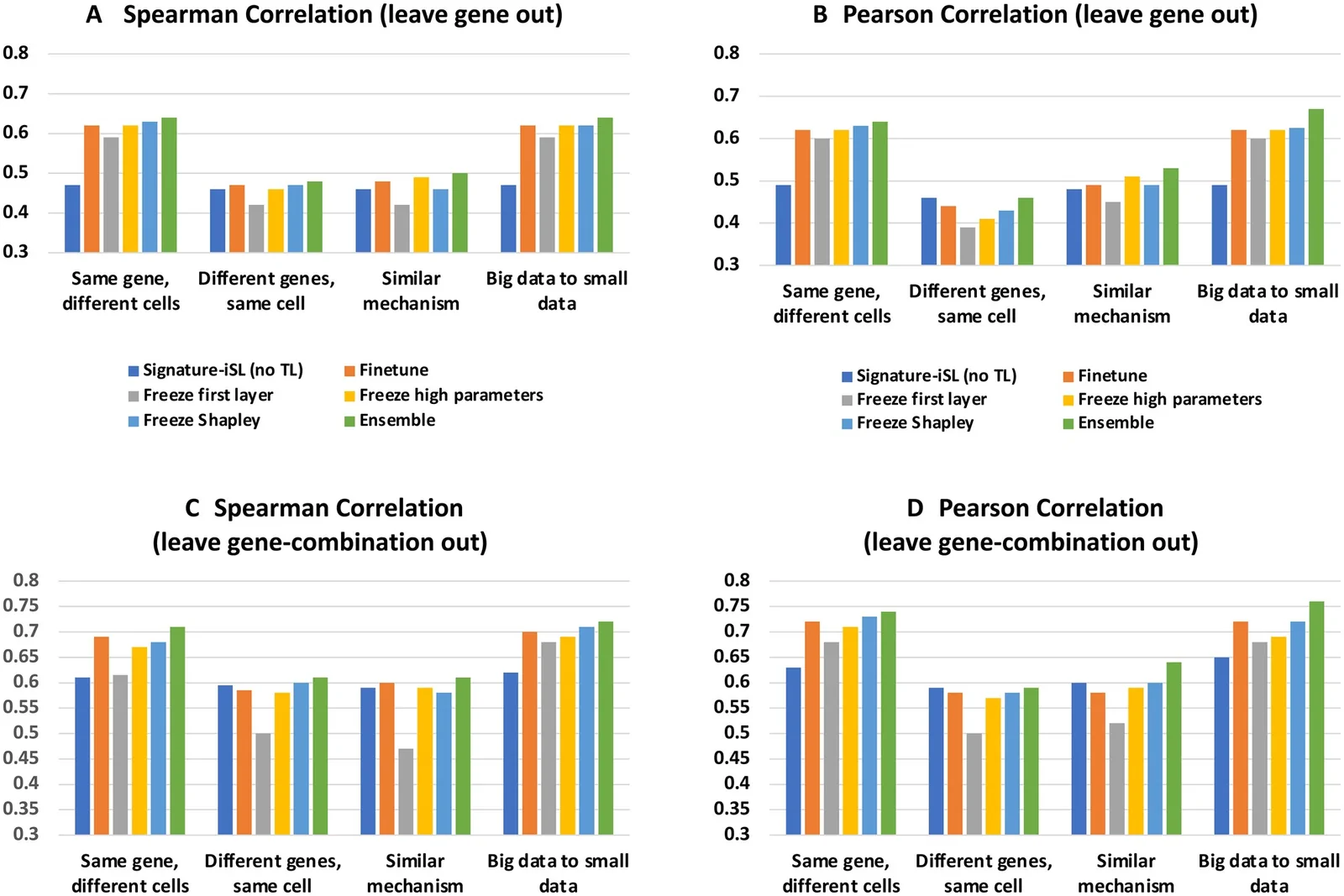
Performance comparison for GCE prediction in (a and b) leave genes out and (c and d) leave gene combinations out settings. Prediction performance is compared between the baseline model, Signature-iGCE, and several TL methods, including finetune, freeze first layer, freeze high parameters, freeze Shapley, and the ensemble of the four. The prediction performance is also compared among four TL scenarios, including same gene and different cells, same cell and different genes, similar mechanism, and big data to small data. To demonstrate SL prediction under the null (no associations), the prediction performance is compared between true labels and randomized labels. (a) Spearman correlation for leave genes out; (b) Pearson correlation for leave genes out; (c) Spearman correlation for leave gene combinations out; and (d) Pearson correlation for leave gene combinations out.

### 3.2 The use of TL methods in TL-iGeneCombo substantially improved SL and GCE prediction using data from the SL knowledge base

Following TL, we compared the performance of the initial models with those of the five TL schemes; finetune, freeze first layer, freeze high parameters, freeze Shapley, and an ensemble of the other four methods. We observed that, on average, TL improved model performances in all metrics for both SL classification and GCE regression tasks in both the leave genes out and leave gene combinations out settings (Figs 2 and 3; Tables 4 and 5, available as supplementary data at *Bioinformatics* online).

For instance, under the same-gene-and-different-cell TL applications in SL prediction (leave genes out in Fig. 2), the average accuracy of Signature-iSL without TL was 65.5%, and the accuracy metric improved with every TL method, reaching 77.0% for finetune, 75.5% for freeze first layer, 78.9% for freeze high parameters, 76.6% for freeze Shapley, and 79.6% for the ensemble. Similarly, under the same-gene-and-different-cell TL application in GCE data (leave genes out in Fig. 3), the average Spearman correlation coefficient of the baseline model without TL was 0.47, and the correlation score improved with each TL model, reaching 0.63 for finetune, 0.59 for freeze first layer, 0.62 for freeze high parameters, 0.62 for freeze Shapley, and 0.64 for the ensemble of methods. In both SL and GCE prediction, the ensemble TL method achieved the optimal performance, which led to a 21% increase in accuracy and 36% increase in Spearman correlation.

Overall, we observed that the ensemble of all four TL methods outperformed each individual method. In the leave genes out setting, TL improved accuracy of SL prediction by 18.2%, AUC-ROC scores by 16.2%, and F1 metrics by 16.4% (Fig. 2; Table 4, available as supplementary data at *Bioinformatics* online). Similarly, the ensemble TL method improved GCE prediction in Pearson correlation by 22.61% and Spearman correlation by 28.42% (Fig. 3; Table 5, available as supplementary data at *Bioinformatics* online).

Compared to that of Signature-iSL, prediction of SL was roughly similar for each individual TL method. Under the accuracy metric across all TL application scenarios, finetune accuracy increased by 15.3%, and the other methods performed similarly—freeze first layer increasing by 13.4%, freeze high parameters by 18.0%, and freeze Shapley by 14.4%. We observed a similar pattern in the Spearman correlation metric in the GCE prediction task, with an accuracy increases of 26.6% using finetune, 17.1% with freeze first layer, 24.1% with freeze high parameters, and 24.8% with freeze Shapley. However, none of the TL methods was consistently better than the others (see Tables 4 and 5, available as supplementary data at *Bioinformatics* online).

### 3.3 GCE prediction was consistently better in the observed CDKO experiment data than its performance in CDKO data under the random permutation, but SL prediction was not

To investigate whether GCE and SL prediction models truly learned the signals from the CDKO data sets, we trained and tested these prediction models on randomly permuted GCE signals and SL labels. Figure 4 compares the prediction performances of both baseline SL/GCE prediction models, either Signature-iSL or Signature-iGCE, and ensemble TL models between true labels and randomized labels. In leave genes out SL prediction, accuracy of both Signature-iSL and ensemble-TL prediction on the true labels was only slightly better than their accuracy on the randomized labels (Fig. 4a; true labels, *P *= .18; randomized labels, 0.01). In the leave gene combinations out setting, Signature-iSL and ensemble-TL prediction accuracy in SL true labels was statistically higher than those in randomized SL labels (true labels, *P *= .0003; randomized labels, *P* < .0001).

**Figure 4:**
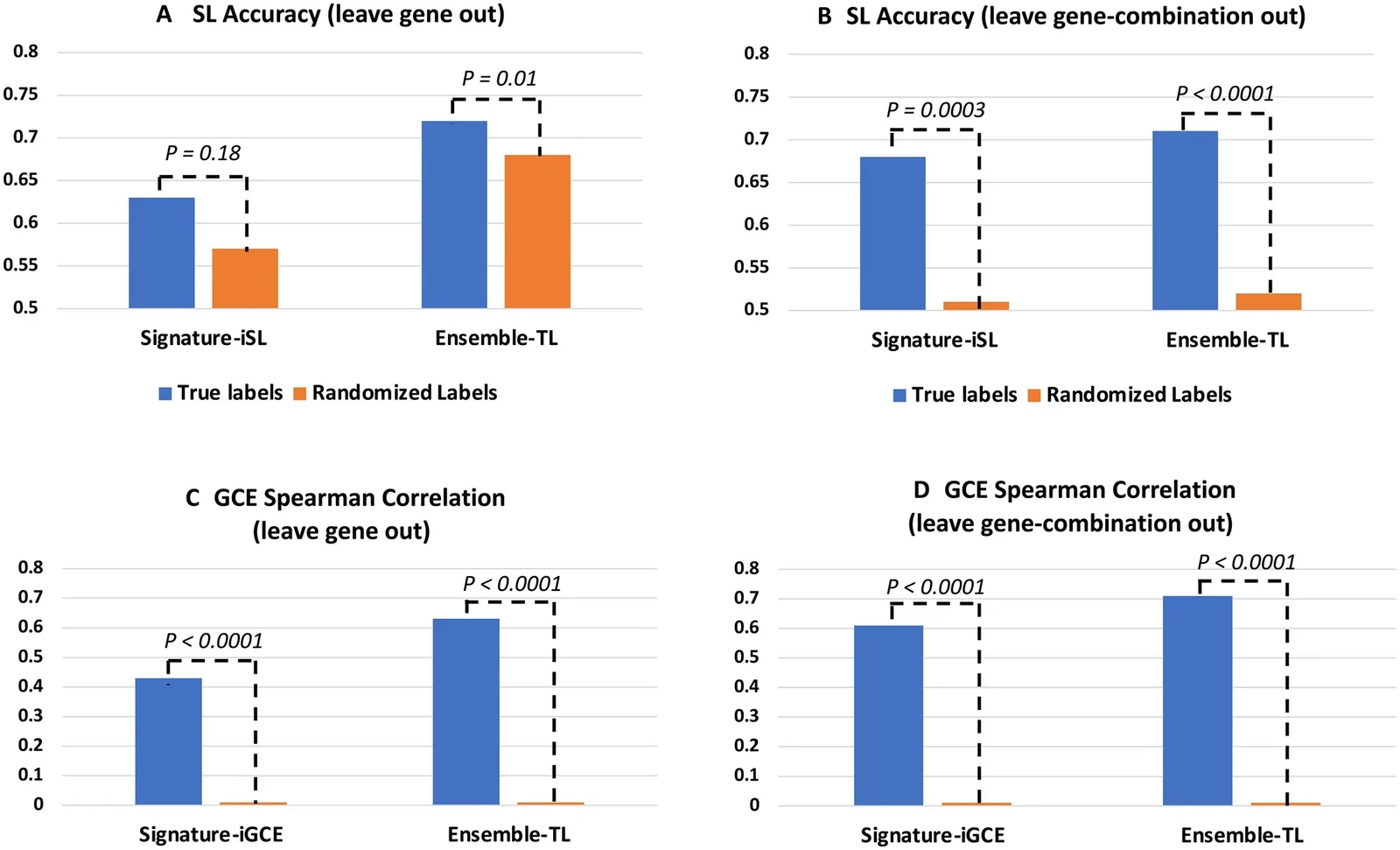
Comparison of SL and GCE between true labels and randomized labels. (a) and (b) comparison of SL prediction accuracy between true and randomized labels in leave genes out and leave gene combinations out. (c) and (d) compare the GCE Spearman correlation between true and randomized labels for leave genes out and leave gene combinations out.

Comparison of GCE prediction on true labels and randomized labels demonstrated much clearer trends. In both the leave genes out and leave gene combinations out settings, both Signature-iGCE and Signature-iSL demonstrated stronger Spearman correlation in predicting true GCE labels than randomized labels (*P *< .0001). Tables 6 and 7, available as supplementary data at *Bioinformatics* online, present the performance of the models in randomized labels.

### 3.4 The difference in SL/GCE prediction performance between source data and target data determined the effectiveness of TL

During the TL, source data did not always improve SL or GCE prediction in the target data. We hypothesized that the effectiveness of TL depended on the differential SL or GCE prediction performance between source data and target data. Therefore, TL was analyzed in two situations, in which SL/GCE prediction performance was better in either (i) source data than target data (source > target) or (ii) target data than source data (target > source). Figure 4, available as supplementary data at *Bioinformatics* online, highlights these analysis results, in which leave genes out was implemented as the validation data and ensemble TL was the selected method. In Fig. 4A, available as supplementary data at *Bioinformatics* online, under the same-gene-and-different-cells setting, TL achieved a larger gain in SL prediction performance in accuracy and AUC-ROC in situation (i) (source > target) than in situation (ii) (target > source), with an accuracy of 0.20 versus 0.15 (*P *< .0001) and an AUC-ROC of 0.13 versus 0.10 (*P *= .0003). This TL advantage in situation (i) over (ii) was even more apparent under the same-cell-and-different-genes setting, see Fig. 4B, available as supplementary data at *Bioinformatics* online.

In predicting GCE, TL showed a similar pattern in situations (i) and (ii) (Fig. 4C and D, available as supplementary data at *Bioinformatics* online). In situation (i), TL demonstrated increased GCE prediction performance of 42% for Spearman correlation and 36% for Pearson correlation. However, in situation (ii), GCE prediction increased only 34% for Spearman correlation and 27% for Pearson correlation. Their *P*-values were both less than .0001. Under the same-cell-and-different-genes setting, TL showed the same trend. More detailed metrics are reported in Tables 8 and 9, available as supplementary data at *Bioinformatics* online.

### 3.5 The application of TL-iGeneCombo significantly improved prediction of GCE but not SL in a model-guided CDKO experiment design in the 22RV1 prostate cancer cell

Two sequential CDKO experiments were performed in the 22RV1 cell, Fig. 5a. In the first, 50 genes in cell-death pathways and all their 1225 gene combinations were tested (Tang *et al.* 2022). From these, we selected SL gene pairs by consensus from seven different SL scores (Gokbag *et al.* 2024), concluding with 88 SL pairings and 1137 non-SL pairs. This CDKO data set was included in the SLKB. Then, an MLEC-iSL model (Fan *et al.* 2024) was built upon the first CDKO experiment data, and it selected 517 SL and 470 non-SL gene pairs for the second CDKO experiment. These 987 gene pairs included 151 genes from the apoptosis, autosis, and mitotic cell-death pathways. Using the same seven SL scores and consensus as in the first CDKO experiment data analysis, we identified 462 SL gene combinations and 525 non-SL pairings.

**Figure 5:**
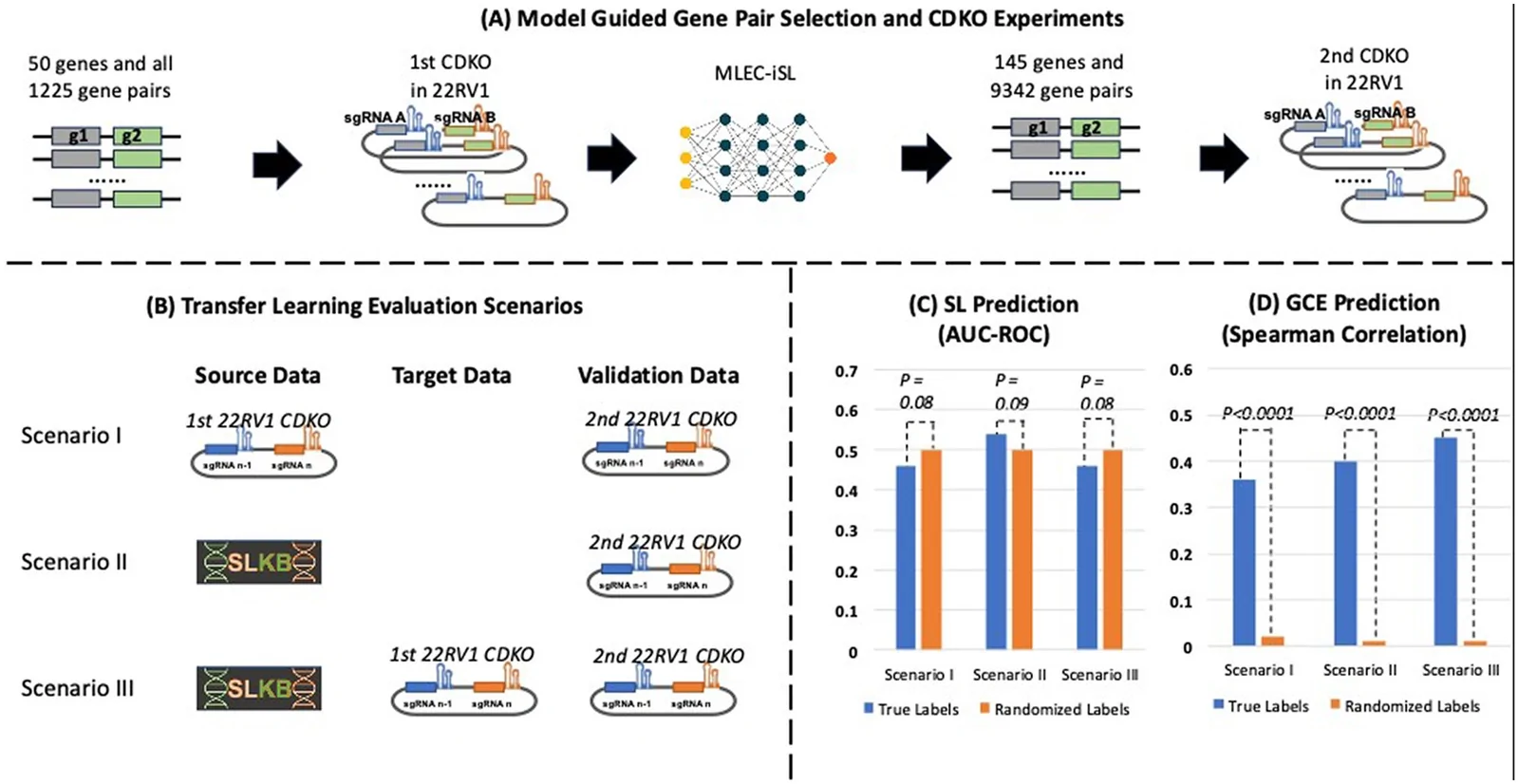
Prospective validation of TL using two sequential rounds of CDKO experiments. (a) The design of two sequential rounds of CDKO experiments in 22RV1 cell. (b) Three validation scenarios are defined. (I) The baseline model was trained in the data of the first-round gene combination double-knockout (CDKO) experiment in the 22RV1 prostate cancer cell and validated in the data of the second-round 22RV1 CDKO experiment. (II) The baseline model was trained in the data of the SLKB) and validated in the second-round 22RV1 CDKO data. (III) Prediction models were trained from the source data (SLKB) to the target data (data of the first-round CDKO experiment in 22RV1) and underwent further validation in the data of the second-round CDKO experiment in 22RV1. (c) SL prediction performance in AUC-R0C values. (d) GCE prediction performance in Spearman correlation.

We designed three validation scenarios to test whether the TL-iGeneCombo method could improve SL or GCE prediction using two sequential rounds of CDKO experiments. Figure 5b presents Scenario I, in which a baseline model was trained in the source data, that of the first-round CDKO experiment in the 22RV1 prostate cancer cell, and was then validated in the data of the second-round 22RV1 CDKO experiment. In Scenario II, the baseline model was trained from eleven sets of CDKO data from the SLKB and validated in the data of the second-round 22RV1 CDKO experiment. Neither Scenario I nor II used TL, but then, in Scenario III, TL was applied to train SL/GCE prediction models from the source data (SLKB CDKO data sets) to the target data (first-round CDKO experiment in 22RV1). These models were further validated in the data of the second-round CDKO experiment in 22RV1. In particular, SL and GCE labels in the second-round CDKO experiment were also randomly permuted (randomized labels) to assess the SL and GCE prediction performance under the null (no associations). In this analysis, we implemented the ensemble TL method.

Figure 5c shows the SL prediction performance, as reflected in AUC-ROC values. Interestingly, SL prediction was not better in true labels than in random labels regardless of whether the model was trained in the data of the first-round 22RV1 CDKO experiment (Scenario I) (*P *= 0.08) or the SLKB (Scenario II) (*P *= 0.09) or by transferring learning from data of the SLKB to data of the first-round 22RV1 CDKO experiment (Scenario III) (*P *= 0.08). However, all three scenarios demonstrated superior performance in predicting GCE, with Spearman correlation ranging from 0.35 to 0.44 in true labels among the three scenarios and about 0.01 to 0.02 in randomized labels (Fig. 5d). The *P*-values for these comparisons were all less than 0.0001. TL showed improved prediction performance in Scenario III (0.44) compared to that of the two baseline models (Scenario I, 0.34; Scenario II, 0.40). Tables 1 and 2, available as supplementary data at *Bioinformatics* online, provide more SL/GCE prediction performance metrics, and these were consistent with the data reported in Fig. 5.

## 4 Discussion

In this paper, we developed a TL framework, TL-iGeneCombo, for SL and GCE prediction for an individual cancer cell. We investigated four TL methods: finetune, freeze first layer, freeze high parameters, and freeze Shapley. An ensemble of the these four methods were also studied. We also studied four TL scenarios–same genes and different cell, same cell and different genes, same mechanism, and big data to small data. In addition, we further compared the TL performance when the source model or target model had better SL/GCE predictions. Using CDKO experiment data collected from the SLKB, we showed that TL generally increased SL and GCE prediction in target data (Figs 2 and 3). None of the four TL methods outperformed the others, but the ensemble of the four yielded the best SL and GCE prediction performance. Considering the four TL scenarios, big-data-to-small-data transfer generally outperformed others (Figs 2 and 3). In analyzing and comparing SL/GCE prediction performance in target data and source data, we showed that TL improved SL/GCE prediction if source data had better prediction than target data, but that was not necessarily the case in reverse (Fig. 4, available as supplementary data at *Bioinformatics* online). Practically, these results suggest how and when TL-iGeneCombo should be implemented. Use of ensemble TL is an optimal approach, and only one SL/GCE source model trained from the SLKB (i.e.source data) is needed. Furthermore, the TL model should be implemented only when the source model has better prediction performance than the target model.

In the prediction of the SL and GCE of new gene combinations, especially when training data and validation shared genes, the application of TL significantly improved prediction compared to that of randomized labels. In the prediction of the SL and GCE of new genes, especially when training data and validation did not share genes, prediction utilizing TL was significantly better than that of the GCE prediction of randomized labels. However, TL improved SL prediction only marginally. This result was also consistent with the prospective training and validation study for TL using two rounds of CDKO experiments on the 22RV1 cell. SL prediction on the second-round CDKO experiment data was no better than randomized-label prediction, but GCE prediction was statistically significantly better than randomized-label prediction. The better SL/GCE prediction in leave gene combinations out than in leave genes out was consistent with those of our MLEC-iSL model (Fan *et al.* 2024) in sample-specific SL prediction and in the pairwise-interaction learning-based graph neural network for the SL model (Liu *et al.* 2022b) for population-based SL prediction. From these results, one important observation is that TL-iGeneCombo did not predict well on SL. It performed no better than the randomized SL label prediction. We believe this was mainly attributable to the inconsistent definition and calculation of SL scores from the CDKO experiments. In our paper introducing the SLKB (Gokbag *et al.* 2024), we showed a mere 1.21% overlap of five SL scores among their top 10% SL gene pairs. In contrast, in the present study, we observed consistently and statistically significantly higher GCE prediction performance on new genes than the prediction with randomized GCE labels, thus supporting the understanding of GCE as a much more robust and informative measurement of gene combination effects from the CDKO experiment data than SL.

The ultimate goal of SL/GCE prediction is to select genes and gene pairs for a new CDKO experiment. In our recently published report of the MLEC-iSL model (Fan *et al.* 2024), we showed that its use improved SL prediction from 3% in our first CDKO experiment to 47% in our second CDKO experiment in the 22RV1 cell. In this paper, we further showed that the model-based selection of gene pairs for our CDKO experiment could improve GCE prediction, but not necessarily SL prediction. However, in the second-round CDKO experiment on the 22RV1 cancer cell, gene pairs were selected based on MLEC-iSL, but not on TL-iGeneCombo. Therefore, direct evidence supporting TL-iGeneCombo–guided selection is still lacking. Ideally, the performance and advantage of GCE or SL prediction by TL-iGeneCombo and gene-pair selection should be tested separately in future independent CDKO experiments. That is, top-ranked genes and gene pairs selected from TL-iGeneCombo-based SL or GCE predictions should be tested in new CDKO experiments before we can conclude whether and how much data from CDKO experiments can improve the performance of TL-iGeneCombo.

Blood tumor cell and solid tumor cell can have different mechanisms. Transferring learning might have better SL or gene combination prediction within each tumor cell type than between two. However, current published CDKO experiments and data may not be able to fully answer this TL question. Among 9 data CDKO experiments presented in Table 1, available as supplementary data at *Bioinformatics* online, only two studies, Han *et al.* 2017 and Horlbeck *et al.* 2018, shared the K562, a leukemia cell line. All the other seven CDKO experiments had solid tumor cells. A direct comparison of TL between these two sets of CDKO experiments, leukemia cell K562 versus other solid tumor cells cannot be conclusive, because they tested different gene sets. Ideally, in both blood tumor cell set and solid tumor cell set, genes or gene combinations shall be consistently selected as source CDKO experiment data and testing CDKO experiment data. This study design will allow us to compare SL and gene combination prediction performance in TL under four conditions: blood cell to blood cell, solid cell to solid cell, blood cell to solid cell, and solid cell to blood cell.

Though SL/GCE prediction showed promising improvement in a target data set when TL-iGeneCombo was applied, the model cannot be used to predict gene combination effect for a cell that has no CDKO data. This is a so-called cold start task. There were several combinationary target selection algorithms developed for this task. For instances, VIPER (Paull *et al.* 2021), OptiCon (Hu *et al.* 2019) within the gene regulatory network, DSCN (Liu *et al.* 2022a), and genome-wide positioning systems network (Cheng *et al.* 2019). These methods, though do not need CDKO experiments as training data, their selected combinatorial targets cannot directly be interpreted as SL gene pairs.

We recently had some success in addressing this cold-start task using a multi-layer encoder prediction model for the prediction of individual sample-specific gene combination effects (MLEC-iGeneCombo) (Shen *et al.* 2025). This model was trained using data from CDKO experiments from many cancer cells at the same time and can predict GCE for a new cancer cell without CDKO data. MLEC-iGeneCombo and TL-iGeneCombo differ primarily by their computational biology models. MLEC-iGeneCombo has several encoders, including a network encoder built on signaling pathways, such as those in the Kyoto Encyclopedia of Genes and Genome (https://www.genome.jp/kegg/) and BioGRID (https://thebiogrid.org), whereas TL-iGeneCombo has a much simpler MLP that uses the signatures derived from the MSigDB **(**Liberzon *et al.* 2011**)**. The multilayer perceptron in TL-iGeneCombo was chosen for its more interpretable structure for TL. In the future, it will be interesting to investigate whether and how GCE/SL in a new cancer cell can be predicted using a more consistent modeling framework than either MLEC-iGeneCombo or TL-iGeneCombo.

## Supplementary Material

btag760_Supplementary_Data

## Data Availability

All developed software can be freely accessed at https://github.com/BirkanGokbag/TL-iGeneCombo  *under GNU General Public License v.3.0*. Alternatively, the software and data can both be accessed at *Zenodo under DOI: 10.5281/zenodo.22889898*.
