# Supplementary material for: TL-iGeneCombo: transfer learning for individual sample specific gene combination effect prediction and selection in cancer cells
Source: Bioinformatics. 2026 Oct 10;42(11):btag760. doi: 10.1093/bioinformatics/btag760 (PMC13744699; doi:10.1093/bioinformatics/btag760)
Supplement: Supplementary file 1 [file btag760_supplementary_data.docx]

**Supplementary Table 1. Selected gene-combination double-knockout (CDKO) experiments from the Synthetic Lethality Knowledge Base (SLKB)**

| **CDKO studies** | **Numbers of genes**  **and gene pairs*** | **Cell lines** | **Gene selection mechanisms** |
| --- | --- | --- | --- |
| Wong et al. 2016 (1) | 48 genes,  1,128 gene pairs | OVCAR8 | Epigenetics regulation in ovarian cancer |
| Han et al. 2017 (2) | 203 genes,  20,387 gene pairs | K562 | **Drug targets** |
| Shen et al. 2017 (3) | 73 genes,  2,628 gene pairs | HeLa, A549 | **Drug targets** include tumor suppressors |
| Horlbeck et al. 2018 (4) | 425 genes,  89,430 gene pairs | Jurkat, K562 | **Non-essential genes** |
| Zhao et al. 2018 (5) | 50 genes,  1,225 gene pairs | A549, HeLa | **Metabolic pathways** |
| Ito et al. 2021 (6) | 3,192 genes,  4,933 paralog pairs | A549, GI-1, HS 936.T, HS 944.T, HSC-5, IPC-298, MEL-202, MEL-JUSO, MeWo, PA-TU-8988S, PK-1 | **Paralog families** |
| Parrish et al. 2021 (7) | 2,000 genes,  1,000 gene pairs | HeLa, PC-9 | **Paralog families** |
| Thompson et al. 2021 (8) | 1,566 genes,  1,125 gene pairs | A-375, MeWo | **Paralog families** |
| Tang et al. 2022 (9) | 50 genes,  1,225 gene pairs | 22Rv1 | **Cell death pathways and drug targets** |

Note: *The numbers indicate full genes and gene pairs used in either gene-combination effect (GCE) or synthetic lethality (SL) prediction tasks. Some genes and gene pairs were filtered because of the absence of omics or gene set profiles, and some were added as a result of the generation of GCE data using the SLKB. Han et al. 2017 (2) was available only for SL prediction tasks due to the lack of dual-sgRNA data for GCE calculation. The large variety of study designs required the simplification of gene-selection mechanisms to carry out transfer learning in the selection of similar mechanisms. As a result, studies grouped under the same selection will be used in the similar-mechanism transfer-learning task, namely drug targets and paralog families***.***

**Supplementary Table 2: Prospective GCE prediction validation**

|  | **Spearman correlation** | **Pearson correlation** |
| --- | --- | --- |
| Scenario I (true labels) | 0.35 | 0.37 |
| Scenario II (true labels) | 0.40 | 0.37 |
| Scenario III (true labels) | 0.45 | 0.51 |
| Scenario I (randomized labels) | 0.03 | 0.04 |
| Scenario II (randomized labels) | 0.01 | 0.01 |
| Scenario III (randomized labels) | 0.01 | 0.01 |

**Supplementary Table 3: Preparation of source and target data for transfer learning**

| **Scenarios** | **CDKO source data**  **and target data sets*** | **Source/target combinations** | **Examples** | |
| --- | --- | --- | --- | --- |
|  |  |  | **Source data** | **Target data** |
| Baseline (no transfer learning) | 1, 2, 3, 4, 5, 6, 7, 8, 9 | 240 SL  230 GCE | N/A | K562 cell in 4 |
| Same genes and different cell | 3, 4, 5, 6, 7, 8, | 6,000 SL  6,000 GCE | Jurkat cell in 4 | K562 cell in 4 |
| Same cell and different genes | 2, 3, 4, 5, 6, 7, 8, | 800 SL  700 GCE | K562 cell in 2 | K562 cell in 4 |
| Similar mechanism | 2, 3, 6, 7, 8 | 900 SL  750 GCE | All cells  in 6 and 7 | A-375 cell in 8 |
| Big data to small data | 1, 2, 3, 4, 5, 6, 7, 8, 9 | 1,200 SL  1,150 GCE | All cells except K562 cell in 4 | K562 cell in 4 |

Note: *The numerals represent the nine CDKO studies: ^1^Wong et al. 2016; ^2^Han et al. 2017; ^3^Shen et al. 2017; ^4^Horlbeck et al. 2018; ^5^Zhao et al. 2018; ^6^Ito et al. 2021; ^7^Parrish et al. 2021; ^8^Thompson et al. 2021; and ^9.^Tang et al. 2022. The **Supplementary Methods** detail each instance of transfer learning.

**Supplementary Table 4: Synthetic lethality (SL) prediction for the true labels**

|  | All applications | Overlapping  gene | Overlapping  cell | Overlapping  mechanism | Big data to small data |
| --- | --- | --- | --- | --- | --- |
| Accuracy (after TL) | '+0.76/+0.73 | '+0.8/+0.74 | '+0.61/+0.67 | '+0.67/+0.7 | '+0.75/+0.72 |
| AUC-ROC (after TL) | '+0.67/+0.8 | '+0.72/+0.81 | '+0.55/+0.72 | '+0.56/+0.75 | '+0.62/+0.8 |
| AUC-ROC@0.20 (after TL) | '+0.72/+0.71 | '+0.72/+0.73 | '+0.73/+0.65 | '+0.73/+0.69 | '+0.74/+0.71 |
| AUC-ROC@0.10 (after TL) | '+0.72/+0.67 | '+0.71/+0.68 | '+0.73/+0.63 | '+0.73/+0.65 | '+0.73/+0.67 |
| F-1 (after TL) | '+0.83/+0.73 | '+0.87/+0.74 | '+0.66/+0.65 | '+0.76/+0.69 | '+0.8/+0.73 |
| Precision (after TL) | '+0.89/+0.73 | '+0.9/+0.74 | '+0.82/+0.69 | '+0.9/+0.71 | '+0.86/+0.72 |
| Precision@20 (after TL) | '+0.96/+0.89 | '+0.97/+0.9 | '+0.88/+0.81 | '+0.95/+0.85 | '+0.93/+0.87 |
| Precision@10 (after TL) | '+0.97/+0.92 | '+0.98/+0.94 | '+0.89/+0.82 | '+0.96/+0.88 | '+0.94/+0.9 |
| Recall (after TL) | '+0.8/+0.74 | '+0.85/+0.75 | '+0.6/+0.63 | '+0.69/+0.7 | '+0.78/+0.75 |
| Precision-Recall (after TL) | '+0.93/+0.81 | '+0.95/+0.83 | '+0.87/+0.75 | '+0.92/+0.78 | '+0.9/+0.81 |
| Accuracy (before TL) | '+0.64/+0.69 | '+0.66/+0.69 | '+0.6/+0.64 | '+0.66/+0.69 | '+0.64/+0.69 |
| AUC-ROC (before TL) | '+0.58/+0.74 | '+0.58/+0.74 | '+0.52/+0.68 | '+0.56/+0.74 | '+0.58/+0.74 |
| AUC-ROC@0.20 (before TL) | '+0.71/+0.68 | '+0.73/+0.68 | '+0.71/+0.65 | '+0.74/+0.68 | '+0.71/+0.68 |
| AUC-ROC@0.10 (before TL) | '+0.71/+0.65 | '+0.73/+0.65 | '+0.72/+0.63 | '+0.74/+0.64 | '+0.71/+0.65 |
| F-1 (before TL) | '+0.71/+0.68 | '+0.74/+0.68 | '+0.66/+0.63 | '+0.75/+0.67 | '+0.71/+0.68 |
| Precision (before TL) | '+0.85/+0.71 | '+0.88/+0.71 | '+0.83/+0.66 | '+0.91/+0.72 | '+0.85/+0.71 |
| Precision@20 (before TL) | '+0.91/+0.83 | '+0.95/+0.83 | '+0.9/+0.77 | '+0.95/+0.83 | '+0.91/+0.83 |
| Precision@10 (before TL) | '+0.94/+0.87 | '+0.97/+0.88 | '+0.92/+0.83 | '+0.97/+0.88 | '+0.94/+0.87 |
| Recall (before TL) | '+0.63/+0.67 | '+0.66/+0.66 | '+0.59/+0.63 | '+0.66/+0.65 | '+0.63/+0.67 |
| Precision-Recall (before TL) | '+0.89/+0.77 | '+0.91/+0.77 | '+0.86/+0.73 | '+0.93/+0.77 | '+0.89/+0.77 |

Note: In the x/y denotation, the numeral x represents the result in leave-gene-out validation, and the numeral y is the result for leave-gene-combination-out validation. AUC-ROC, area under the receiver-operating characteristic curve; TL, transfer learning

**Supplementary Table 5: Gene-combination effect (GCE) prediction for the true labels**

|  | All applications | Overlapping  gene | Overlapping  cell | Overlapping  mechanism | Big data to small data |
| --- | --- | --- | --- | --- | --- |
| Spearman correlation  (after TL) | '+0.62/+0.7 | '+0.64/+0.71 | '+0.47/+0.61 | '+0.5/+0.6 | '+0.65/+0.73 |
| Pearson correlation  (after TL) | '+0.63/+0.73 | '+0.65/+0.75 | '+0.46/+0.59 | '+0.52/+0.64 | '+0.68/+0.76 |
| Spearman correlation (before TL) | '+0.48/+0.63 | '+0.47/+0.62 | '+0.45/+0.59 | '+0.45/+0.57 | '+0.48/+0.63 |
| Pearson correlation  (before TL) | '+0.51/+0.66 | '+0.49/+0.64 | '+0.45/+0.58 | '+0.48/+0.6 | '+0.51/+0.66 |

Note: In the x/y denotation, the numeral x represents the result in leave-gene-out validation, and the numeral y is the result for leave-gene-combination-out validation. TL, transfer learning

**Supplementary Table 6: Synthetic lethality (SL) prediction for the randomized labels**

|  | All applications | Overlapping  gene | Overlapping  cell | Overlapping  mechanism | Big data to small data |
| --- | --- | --- | --- | --- | --- |
| Accuracy (after TL) | '+0.7/+0.5 | '+0.74/+0.5 | '+0.54/+0.49 | '+0.63/+0.5 | '+0.69/+0.5 |
| AUC-ROC (after TL) | '+0.45/+0.5 | '+0.47/+0.5 | '+0.41/+0.48 | '+0.4/+0.5 | '+0.42/+0.5 |
| AUC-ROC@0.20 (after TL) | '+0.58/+0.5 | '+0.56/+0.5 | '+0.63/+0.5 | '+0.63/+0.51 | '+0.6/+0.51 |
| AUC-ROC@0.10 (after TL) | '+0.59/+0.5 | '+0.57/+0.5 | '+0.64/+0.51 | '+0.64/+0.51 | '+0.61/+0.51 |
| F-1 (after TL) | '+0.79/+0.5 | '+0.84/+0.5 | '+0.6/+0.45 | '+0.73/+0.49 | '+0.75/+0.51 |
| Precision (after TL) | '+0.85/+0.5 | '+0.87/+0.5 | '+0.75/+0.48 | '+0.86/+0.5 | '+0.8/+0.5 |
| Precision@20 (after TL) | '+0.84/+0.49 | '+0.86/+0.49 | '+0.72/+0.48 | '+0.85/+0.51 | '+0.79/+0.51 |
| Precision@10 (after TL) | '+0.84/+0.49 | '+0.87/+0.49 | '+0.7/+0.45 | '+0.86/+0.52 | '+0.79/+0.5 |
| Recall (after TL) | '+0.76/+0.51 | '+0.82/+0.51 | '+0.55/+0.45 | '+0.66/+0.49 | '+0.73/+0.53 |
| Precision-Recall (after TL) | '+0.86/+0.52 | '+0.88/+0.51 | '+0.8/+0.54 | '+0.88/+0.52 | '+0.82/+0.53 |
| Accuracy (before TL) | '+0.58/+0.5 | '+0.59/+0.5 | '+0.54/+0.5 | '+0.6/+0.51 | '+0.58/+0.5 |
| AUC-ROC (before TL) | '+0.42/+0.5 | '+0.4/+0.5 | '+0.4/+0.5 | '+0.39/+0.5 | '+0.42/+0.5 |
| AUC-ROC@0.20 (before TL) | '+0.6/+0.51 | '+0.61/+0.51 | '+0.61/+0.51 | '+0.63/+0.5 | '+0.6/+0.51 |
| AUC-ROC@0.10 (before TL) | '+0.61/+0.51 | '+0.62/+0.51 | '+0.62/+0.52 | '+0.64/+0.51 | '+0.61/+0.51 |
| F-1 (before TL) | '+0.66/+0.48 | '+0.68/+0.48 | '+0.61/+0.48 | '+0.71/+0.48 | '+0.66/+0.48 |
| Precision (before TL) | '+0.79/+0.5 | '+0.81/+0.49 | '+0.75/+0.49 | '+0.85/+0.5 | '+0.79/+0.5 |
| Precision@20 (before TL) | '+0.77/+0.5 | '+0.8/+0.5 | '+0.73/+0.5 | '+0.84/+0.5 | '+0.77/+0.5 |
| Precision@10 (before TL) | '+0.77/+0.48 | '+0.8/+0.47 | '+0.73/+0.46 | '+0.85/+0.47 | '+0.77/+0.48 |
| Recall (before TL) | '+0.59/+0.49 | '+0.61/+0.48 | '+0.55/+0.49 | '+0.63/+0.46 | '+0.59/+0.49 |
| Precision-Recall (before TL) | '+0.81/+0.54 | '+0.83/+0.53 | '+0.79/+0.55 | '+0.87/+0.53 | '+0.81/+0.54 |

Note: In the x/y denotation, the numeral x represents the result in leave-gene-out validation, and the numeral y is the result for leave-gene-combination-out validation. AUC-ROC, area under the receiver-operating characteristic curve; TL, transfer learning

**Supplementary Table 7: Gene-combination effect (GCE) prediction for the randomized labels**

|  | All applications | Overlapping  gene | Overlapping  cell | Overlapping  mechanism | Big data to  small data |
| --- | --- | --- | --- | --- | --- |
| Spearman correlation  (after TL) | '0.0/0.0 | '0.0/+0.01 | '+0.01/-0.0 | '0.0/+0.01 | '-0.0/0.0 |
| Pearson correlation  (after TL) | '0.0/+0.01 | '0.0/+0.01 | '+0.01/-0.0 | '-0.0/+0.01 | '-0.0/+0.01 |
| Spearman correlation  (before TL) | '0.0/+0.01 | '0.0/+0.01 | '+0.01/0.0 | '-0.0/+0.01 | '0.0/+0.01 |
| Pearson correlation  (before TL) | '0.0/+0.01 | '0.0/+0.01 | '+0.01/0.0 | '0.0/+0.01 | '0.0/+0.01 |

Note: In the x/y denotation, the numeral x represents the result in leave-gene-out validation, and the numeral y is the result for leave-gene-combination-out validation. TL, transfer learning

**Supplementary Table 8: Average percentage of impact on performance of ensemble transfer learning when the source model outperformed the target model.**

| Synthetic lethality (SL) classification task | | | | | |
| --- | --- | --- | --- | --- | --- |
|  | All  applications | Same gene and different cells | Same cell and different genes | Similar  mechanism | Big data to  small data |
| Accuracy | +14.75/+3.95 | +20.13/+8.03 | +10.32/+7.39 | +2.82/+1.14 | +17.95/+5.29 |
| AUC-ROC | +11.65/+5.61 | +12.33/+9.97 | +15.74/+7.21 | +2.15/+1.31 | +6.9/+7.83 |
| F-1 | +13.16/+5.19 | +17.33/+11.29 | +4.26/+5.0 | +2.3/+2.75 | +13.15/+7.64 |
| Precision | +3.44/+1.43 | +2.75/+4.03 | +2.45/+6.31 | +2.18/-0.64 | +1.42/+1.44 |
| Recall | +20.99/+7.46 | +28.55/+17.31 | +10.68/+2.5 | +2.89/+5.13 | +23.22/+12.33 |
| Precision-Recall | +3.23/+3.68 | +3.77/+7.2 | -0.04/+3.11 | +0.62/+0.26 | +1.39/+4.73 |
| Gene-combinatorial effect (GCE) regression task | | | | | |
|  | All  applications | Same gene and different cells | Same cell and different genes | Similar  mechanism | Big data to  small data |
| Spearman correlation | +23.91/+9.25 | +42.32/+16.14 | +2.85/+1.08 | +6.82/+4.26 | +37.84/+17.21 |
| Pearson correlation | +21.1/+10.61 | +36.19/+17.71 | +1.72/-2.24 | +3.66/+4.61 | +35.46/+16.47 |
| Cosine similarity | +19.24/+9.62 | +31.33/+15.49 | +5.02/-4.88 | +3.69/+4.35 | +31.57/+14.89 |
| Mean absolute error | -16.53/-12.03 | -24.42/-19.13 | -4.61/+3.08 | -2.69/-2.95 | -16.87/-12.8 |

Note: In the x/y denotation, x represents the percentage of change in the leave-gene-out setting, and y indicates the percentage of change in the leave-gene-combination-out setting. For SL and GCE predictions, we used F-1 and Spearman correlation metrics to identify higher-scoring source models. AUC-ROC, area under the receiver-operating characteristic curve

**Supplementary Table 9: Average percentage of impact on performance of ensemble transfer learning when the target model outperformed the source model.**

| Synthetic lethality classification task | | | | | |
| --- | --- | --- | --- | --- | --- |
|  | All  applications | Same genes and different cell | Same cell and different genes | Similar mechanism | Big data to  small data |
| Accuracy | +13.64/+3.84 | +14.67/+3.23 | -4.66/+1.74 | -25.0/N/A | N/A |
| AUC-ROC | +10.66/+5.72 | +9.61/+5.35 | -3.4/+3.46 | N/A | N/A |
| F-1 | +10.35/+5.22 | +11.71/+4.69 | -5.64/+0.96 | -14.29/N/A | N/A |
| Precision | +0.24/+0.36 | +0.29/-0.85 | -1.47/+2.18 | N/A | N/A |
| Recall | +18.92/+8.67 | +21.42/+8.8 | -8.5/-0.72 | -25.0/N/A | N/A |
| Precision-Recall | +2.51/+3.79 | +2.74/+3.71 | +1.06/+1.24 | N/A | N/A |
| Gene-combinatorial effect regression task | | | | | |
|  | All  applications | Same genes and different cell | Same cell and different genes | Similar mechanism | Big data to  small data |
| Spearman correlation | +29.23/+10.91 | +33.69/+13.31 | +6.45/+4.56 | N/A/-0.19 | -2.74/-0.11 |
| Pearson correlation | +22.17/+10.66 | +26.81/+13.72 | -0.83/+1.47 | N/A/+0.4 | -2.13/+1.4 |
| Cosine similarity | +18.39/+9.0 | +22.31/+12.17 | -4.75/-1.47 | N/A/+0.39 | -2.75/+1.57 |
| Mean absolute error | -13.09/-9.7 | -17.0/-14.3 | +3.3/+0.96 | N/A/+7.36 | +7.06/-0.98 |

Note: In the x/y denotations, x represents the percentage of change in the leave-gene-out setting, and y is the percentage of change in the leave-gene-combination-out split settings. For SL and GCE predictions, we used F-1 and Spearman correlation metrics to identify higher-scoring target models. N/A, not available, no selection was possible. AUC-ROC, area under the receiver-operating characteristic curve

**Supplementary Table 10: Prospective synthetic lethality (SL) prediction validation**

|  | Accuracy | AUC-ROC | | | AUPR | F-1 | Precision | | | Recall |
| --- | --- | --- | --- | --- | --- | --- | --- | --- | --- | --- |
|  |  | All | @20 | @10 |  |  | All | @20 | @10 |  |
| Scenario I  (true labels) | 0.49 | 0.46 | 0.51 | 0.51 | 0.46 | 0.35 | 0.44 | 0.48 | 0.52 | 0.29 |
| Scenario II  **(true labels)** | 0.53 | 0.54 | 0.51 | 0.50 | 0.50 | 0.51 | 0.50 | 0.51 | 0.46 | 0.51 |
| Scenario III  **(true labels)** | 0.47 | 0.46 | 0.49 | 0.50 | 0.45 | 0.41 | 0.43 | 0.42 | 0.41 | 0.37 |
| Scenario I (randomized labels) | 0.51 | 0.50 | 0.50 | 0.50 | 0.48 | 0.37 | 0.46 | 0.47 | 0.47 | 0.31 |
| Scenario II (randomized labels) | 0.50 | 0.50 | 0.51 | 0.50 | 0.48 | 0.47 | 0.47 | 0.50 | 0.49 | 0.48 |
| Scenario III (randomized labels) | 0.50 | 0.49 | 0.50 | 0.50 | 0.47 | 0.44 | 0.47 | 0.46 | 0.45 | 0.41 |

Note: The AUC-ROC@X indicates the area under the curve values calculated under the false-positive rate X. Precision@X indicates the precision metrics calculated within the top X% of the predicted highest-probability gene pairs. AUC-ROC, area under the receiver-operating characteristic curve; AUPR, area under the precision-recall curv

**Supplementary Figure 1**

**Supplementary Figure 2**

**Supplementary Figure 3**

**Supplementary Figure 4**

# Supplementary Methods

## 1 Gradient calculations for Signature-iSL

During forward propagation, each layer returns a vector according to their sizes, named similarly with respect to layer names. Let $I=\left( I_{1},\ldots,I_{3021} \right)$ denote the row vector of features generated from gene pairs; their molecular signatures and multi-omics data that are fed into the model. Let $w_{M}=\left\{ w_{H_{1}}, w_{H_{2}}, w_{O} \right\}$ and $B_{M}=\left\{ B_{H_{1}}, B_{H_{2}}, B_{O} \right\}$ denote the set of parameter weights and biases for each respective layer, excluding the input layer as it does not contain any trainable parameters in our design. Due to fully connected layer design, notice that each layer parameter takes the following shape: $w_{H_{1}}=3021 \times256$, $w_{H_{2}}=256 \times128$, and $w_{O}=128 \times1$. Considering bias, dropout, and activation functions, forward propagation is carried out within the layers as the following:

|  | $H_{1}=LeakyReLU(Dropout(I^{T}\otimes w_{H_{1}}+B_{H_{1}}, 20\%))$ | (1‑1) |
| --- | --- | --- |

|  | $H_{2}=LeakyReLU(Dropout(H_{1}^{T}\otimes w_{H_{2}}+B_{H_{2}}, 20\%))$ | (1‑2) |
| --- | --- | --- |

|  | $O=\left\{ \begin{aligned} Sigmoid\left( H_{2}^{T}\otimes w_{O}+B_{O} \right) \\ H_{2}^{T}\otimes w_{O}+B_{O} \end{aligned} \right. {if SL \atop if GCE}$ | (1‑3) |
| --- | --- | --- |

To discuss how each TL method impacts model training, let us define a few more concepts in detail. Let $L_{BCE}$ and $L_{MAE}$ be the loss functions for BCE and MAE respectively, taking in parameters *O* and *y* such that *O* is the output layer’s prediction and *y* is the data label. Let $G_{LRU}$ and $G_{Sigmoid}$define the gradients for LeakyReLU and sigmoid activation functions. Let us also define the gradients for each layer of the model as $G_{M}=\left\{ G_{H_{1}}, G_{H_{2}}, G_{O} \right\}$. For simplicity, we do not include dropout effect in gradient calculation as they will be simplified in the process. We can calculate gradients of each variable as follows where *x* is a random parameter in each layer.

### 1.1 Leaky ReLU activation

|  | $LeakyReLU(x)=\left\{ \begin{aligned} x \\ 0.01\times x \end{aligned} \right. {\mathrm{if}x \geq0 \atop\mathrm{otherwise}}$ | (1‑4) |
| --- | --- | --- |

|  | $G_{LRU}(x)=\frac{\partial LeakyReLU(x)}{\partial x}=\left\{ \begin{aligned} 1 \\ 0.01 \end{aligned} \right. {\mathrm{if}x \geq0 \atop\mathrm{otherwise}}$ | (1‑5) |
| --- | --- | --- |

### 1.2 Sigmoid activation

|  | $Sigmoid(x)=\frac{1}{1+e^{-x}}$ | (1‑6) |
| --- | --- | --- |

|  | $G_{Sigmoid}(x)=\frac{\partial Sigmoid(x)}{\partial x}=Sigmoid(x)\times(1- Sigmoid(x))$ | (1‑7) |
| --- | --- | --- |

### 1.3 Binary cross entropy loss

|  | $L_{BCE}(O,y)=- y\times\log\left( O \right)-(1-y)\times\log\left( 1-O \right)$ | (1‑8) |
| --- | --- | --- |

|  | $\frac{\partial L_{BCE}(O,y)}{\partial Sigmoid(O)}=-\frac{y}{O}+\frac{1-y}{1-O}$ | (1‑9) |
| --- | --- | --- |

|  | $\frac{\partial L_{BCE}(O,y)}{\partial O}=\frac{\partial L_{BCE}(O,y)}{\partial Sigmoid(O)}\times\frac{\partial Sigmoid(O)}{\partial O}= O-y$ | (1‑10) |
| --- | --- | --- |

### 1.4 Mean absolute error

|  | $L_{MAE}(O,y)=\left\vert O-y \right\vert$ | (1‑11) |
| --- | --- | --- |

|  | $\frac{\partial L_{MAE}(O,y)}{\partial O}=\left\{ \begin{aligned} +1 \mathrm{if} O>y \\ -1 \mathrm{if} O<y \\ \approx0 o.w. \end{aligned} \right.$ | (1‑12) |
| --- | --- | --- |

### 1.5 Generalizing gradient of loss functions for SL and GCE prediction tasks

| $\frac{\partial L}{\partial O}=\left\{ \begin{aligned} \frac{\partial L_{BCE}(O,y)}{\partial O} \\ \frac{\partial L_{MAE}(O,y)}{\partial O} \end{aligned} \right.{if SL \atop if GCE}$ | (1‑13) |
| --- | --- |

### 1.6 Gradient calculations for layer weights and biases

|  | $G_{W_{O}}=\frac{\partial L}{\partial w_{o}}=\frac{\partial L}{\partial O}\times\frac{\partial O}{\partial w_{o}}$ | (1‑14) |
| --- | --- | --- |

|  | $G_{B_{O}}=\frac{\partial L}{\partial B_{o}}=\frac{\partial L}{\partial O}\times\frac{\partial O}{\partial B_{o}}$ | (1‑15) |
| --- | --- | --- |

|  | $G_{W_{H_{2}}}=\frac{\partial L}{\partial w_{H_{2}}}=\frac{\partial L}{\partial O}\times\frac{\partial O}{\partial LeakyReLU(H_{2})}\times\frac{\partial LeakyReLU(H_{2})}{\partial H_{2}}\times\frac{\partial H_{2}}{\partial w_{H_{2}}}$ | (1‑16) |
| --- | --- | --- |

|  | $G_{B_{H_{2}}}=\frac{\partial L}{\partial B_{H_{2}}}=\frac{\partial L}{\partial O}\times\frac{\partial O}{\partial LeakyReLU(H_{2})}\times\frac{\partial LeakyReLU(H_{2})}{\partial H_{2}}\times\frac{\partial H_{2}}{\partial B_{H_{2}}}$ | (1‑17) |
| --- | --- | --- |

|  | $G_{{W_{H}}_{1}}=\frac{\partial L}{\partial w_{H_{1}}}=\frac{\partial L}{\partial O}\times\frac{\partial O}{\partial LeakyReLU(H_{2})}\times\frac{\partial LeakyReLU(H_{2})}{\partial H_{2}}\times\frac{\partial H_{2}}{\partial LeakyReLU(H_{1})}\times\frac{\partial LeakyReLU(H_{1})}{\partial H_{1}}\times\frac{\partial H_{1}}{\partial w_{H1}}$ | (1‑18) |
| --- | --- | --- |

|  | $G_{B_{H_{1}}}=\frac{\partial L}{\partial B_{H_{1}}}=\frac{\partial L}{\partial O}\times\frac{\partial O}{\partial LeakyReLU(H_{2})}\times\frac{\partial LeakyReLU(H_{2})}{\partial H_{2}}\times\frac{\partial H_{2}}{\partial LeakyReLU(H_{1})}\times\frac{\partial LeakyReLU(H_{1})}{\partial H_{1}}\times\frac{\partial H_{1}}{\partial B_{H1}}$ | (1‑19) |
| --- | --- | --- |

Notice that the terms simplify in a nice manner due to neural networks’ matrix multiplication during training. First, the derivatives of LeakyReLU will yield a constant for each model parameter based on their value. Second, the rate of change between a layer’s input and output can be simplified to that layer’s parameters (e.g., $\frac{\partial O}{\partial H_{2}}=w_{o}$). Third, the derivative of a layer’s output with respect to the layer’s parameters will yield the input to that layer (e.g., $\frac{\partial O}{\partial w_{o}}=H_{2}$). Fourth, bias terms’ gradients with respect to their outputs in hidden layer simplify to 1 unlike weight parameters that simplify to the input to that given layer, this is because bias addition is independent of the input. Finally, dropout regularization does not impact gradient calculation but rather prevents a select percentage of parameters from training in each applied layer, in this study dropout is set to 20% and can be treated as a constant. Then, each gradient can be simplified to the following with appropriate matrix multiplications:

|  | $G_{W_{o}}=\frac{\partial L}{\partial w_{o}}=\frac{\partial L}{\partial o}\times H_{2}$ | (1‑20) |
| --- | --- | --- |

|  | $G_{B_{o}}=\frac{\partial L}{\partial B_{o}}=\frac{\partial L}{\partial o}$ | (1‑21) |
| --- | --- | --- |

|  | $G_{W_{H_{2}}}=\frac{\partial L}{\partial w_{H_{2}}}=\frac{\partial L}{\partial o}\times w_{o}\times H_{1}$ | (1‑22) |
| --- | --- | --- |

|  | $G_{B_{H_{2}}}=\frac{\partial L}{\partial B_{H_{2}}}=\frac{\partial L}{\partial o}\times w_{o}$ | (1‑23) |
| --- | --- | --- |

|  | $G_{w_{H_{1}}}=\frac{\partial L}{\partial w_{H_{1}}}=\frac{\partial L}{\partial o}\times w_{o}\times w_{H_{2}}\times I$ | (1‑24) |
| --- | --- | --- |

|  | $G_{B_{H_{1}}}=\frac{\partial L}{\partial B_{H_{1}}}=\frac{\partial L}{\partial o}\times w_{o}\times w_{H_{2}}$ | (1‑25) |
| --- | --- | --- |

Furthermore, let us also define the Jacobian matrix that stores the gradients. During TL, each calculated gradient will be impacted differently based on whichever TL method is carried out. For a layer *H* that takes an input of size *N* and returns a size of *M*, loss function *L*, and parameter weights $w_{H}$, the Jacobian matrix for the layer’s weight parameters take the following form:

|  | $G_{H}=\left[ \begin{matrix} \frac{\partial L}{\partial w_{H}(1,1)} & \cdots& \frac{\partial L}{\partial w_{H}(1,M)} \\ \vdots& \ddots& \vdots\\ \frac{\partial L}{\partial w_{H}(N,1)} & \cdots& \frac{\partial L}{\partial w_{H}(N,M)} \end{matrix} \right]$ | (1‑26) |
| --- | --- | --- |

### 1.7 Parameter freezing implementation

PyTorch library was used again for each TL method. Implementation wise, PyTorch allows gradient calculations to be disabled for all parameters in a layer by setting the ‘requires_grad’ parameter of a layer to Boolean false. Computationally, there is no out of the box implementation for disabling only specific parameters within a layer. In order to allow such specific freezing, the indices of parameters were identified such that freezing was implemented using a mask. Using the masks, we restored the original weights back to source weights in each iteration, effectively freezing the weights. As a result, frozen parameters did not change but still contribute to model prediction.

### 1.8 Hyperparameter Selection

All models, both in standard deep learning and transfer learning tasks, were developed in PyTorch v2 framework (10). Each model used Adamax optimizer (11) in its design with learning rates 1e-2, 1e-3, and 1e-4, and the best performing learning rate is chosen through minimum loss in grid search against held-out validation datasets. Each model was trained for 1,000 iterations with early stopping; if validation loss did not decrease after 50 iterations, the weights of best performing model so far were retained. Batch size for classification tasks was set to 32 samples whereas batch size for regression tasks was set to 10% of the available training data samples; this is due to sample sparsity of SL classification datasets and abundance of GCE regression datasets. For classification tasks, the number of non-SL gene pairs were down sampled to match the sample size of SL gene pairs. To prevent any potential performance bias caused by data splits, a total of five computational runs were carried out with each accompanied by a different data split. Binary cross entropy (BCE) and mean absolute error (MAE) loss functions were used for classification and regression tasks respectively. MAE was chosen over mean square error (MSE) due to [-1, 1] label range in GCE prediction. At the end of each model’s training, all training parameters, performance metrics, and hyperparameter settings were saved and can be loaded for future inference.

## 2. Shapley method

## 2.1 Present work on Shapley value calculation in deep learning models

Shapley values allow machine learning models to be interpretable and explainable; identifying feature contributions to predictions and as a result understanding how features affect the data and model (12). Shapley value calculations are applicable to many kinds of machine learning models, such as classifiers and regressors through decision trees, random forests, and so forth. Recently, Shapley value calculations have also been applied to deep learning models through a variety of approaches, such as perturbation-based forward propagation and backpropagation-based methods (13). Considering the black box nature of deep learning models, high dimensionality datasets, and large number of data available, Shapley value calculations are complex for deep learning models.

Recently, two Shapley value calculation methods have been developed to address limitations in deep learning space: DeepLIFT and G-DeepShap (13, 14). DeepLIFT is a backpropagation-based approach that calculates Shapley values by looking at the difference between query reference data outputs with respect to query and reference inputs. The reference data is defined to be a ‘default’ state. For instance, to calculate Shapley values for an image dataset, the reference may consist of black images such that each pixel has the value of 0 at each input channel or an average representation of all images in the dataset. Shapley values are calculated by adding features randomly to a coalition of features and taking their prediction difference against reference dataset. DeepLIFT consists of three rules: linear rule for dense and convolutional layers, rescale rule for activation functions, and reveal cancel rule for correcting misleading gradients for Shapley calculations. G-DeepShap also is a backpropagation-based approach and builds on DeepLIFT’s theory. G-DeepShap extends Shapley value calculations to other frameworks, such as decision tree models, and introduces new rules such as taking a sampling approach for designing reference datasets rather than using the same reference dataset for all calculations.

Both frameworks are similar in two main aspects: each (1) utilizes a separate reference dataset in their Shapley calculation, and (2) incorporates sampling on feature coalitions. Sampling process is crucial in high dimensionality data and effectively follows Monte Carlo sampling, where the sampled simulations exhibit the underlying distribution of the entire dimensions (15). However, both approaches are not suitable for SL or GCE tasks due to one aspect: the use of references data that Shapley values are calculated on, and feature coalition generation for Shapley value calculation. First, reference datasets do not exist for SL or GCE data where molecular signature co-membership and multi-omics of gene pairs are feature definitions. Biologically, an empty vector would not explain a gene pair’s features and cannot be used as reference. Secondly, adding gene features one at a time would not biologically explain the actual data at hand as gene pairs cover a high variety of molecular signatures. Doing so would be under the assumption that molecular signatures have independent effects from each other, which is not the case from a systems biology standpoint. As a result, these approaches are not suitable for SL or GCE Shapley value calculations. We devised our own Shapley value calculation algorithm by also following Monte Carlo sampling theory.

## 2.2 Monte Carlo based Shapley value calculation

Monte Carlo simulation employs random sampling techniques to estimate the underlying distribution, particularly useful in computationally expensive tasks. One of the employed areas of Monte Carlo is in calculation of Shapley values in machine learning tasks, where contribution of each feature to the feature coalition is obtained (16). Shapley values are originally estimated through all possible coalitions of features which result in an exponential number of runs to estimate all values (i.e., $N$ features result in $2^{N}$ possible combinations for our molecular signature features). Monte Carlo sampling (MCS) is one of the ways Shapley values can be estimated through a low computational cost, where coalitions are sampled from all possible combinations. Based on the law of large numbers, Monte Carlo sampling will converge on the distribution of the underlying population after large number of samples (15). As a result, Shapley value estimation with Monte Carlo sampling will also converge to the underlying feature contribution within the entire dataset.

## 2.3 Problem definition for Shapley value calculation

In the context of synthetic lethality (SL) and gene combination effects (GCE), there are three main difficulties in estimating Shapley values for the developed deep learning tasks. First, the large number of features that are used in feature engineering. As the developed models use large number of features, in this case high number of features, Shapley value computation becomes computationally expensive. Second, the data samples consist of gene pairs and their SL categorization or GCE score. Each datapoint is biologically unique and cannot be generalized to all available samples, thus the computation becomes increasingly expensive in large datasets. Third, in the context of molecular signature gene set memberships of a gene pair, gene pair features are mostly sparse as there are many different biological functions and two genes cannot cover more than a few, thereby being sparse. In our experiments, 85.74% of the features were sparse.

## 2.4. Proposed Monte Carlo simulation for Shapley value calculation

We propose a Shapley value calculation algorithm that incorporates MCS and data sampling specifically for SL and GCE datasets using molecular signature data as features. Existing MCS for Shapley value calculation algorithm (**Supplementary Figure 5**) and our proposed solution algorithm (**Supplementary Figure 6**) are illustrated below.In SL classification task, positive and negative Shapley values indicate SL and non-SL contribution respectively. Inversely, in GCE regression task, positive and negative Shapley values indicate an increase in cell viability and decrease in cell viability respectively. Thus, positive Shapley values in SL and negative Shapley values in GCE represent the features that contribute to cell depletion.

**Supplementary Figure 5**. Generalized Monte Carlo sampling for Shapley value calculation

Existing Monte Carlo based Shapley value algorithms calculate Shapley values by sampling over possible combinations of feature coalitions that build over time (**algorithm 1, line 4**). In each sampling iteration, feature coalition is created randomly to allow estimation of each feature impact over a normal distribution (**algorithm 1, line 6**). Each feature is added one at a time, and at each step the trained model’s prediction with and without that gene in coalition is calculated (**algorithm 1, line 9**). That difference is then averaged over input data samples in **line 10**, and later across all sampling iterations in **line 14**.

**Supplementary Figure 6**. Proposed Monte Carlo sampling for Shapley value calculation in SL/GCE models

Our proposed Monte Carlo based Shapley value calculation method does not rely on sampling and random generation of a coalition of features in its design (**Supplementary Figure 6**). Instead, the algorithm starts by identifying gene pairs that participate in a molecular signature gene set and obtains those specific samples (**algorithm 2, lines 5 and 6**). Then, for the same gene pairs, the select feature is set to null for all the samples (**algorithm 2, lines 7 and 8**). Finally, their difference is obtained and later averaged to obtain Shapley values of the select feature (**algorithm 2, lines 9 and 10**). The motivation behind is approach is that the dataset is not synthetically perturbed where each feature is added one at a time, but instead Shapley value is calculated from gene pairs that specifically exhibit that specific feature. If a gene set is not present in genes A and B, it shouldn’t be used to calculate Shapley values on the gene pair A and B. However, in the existing approaches, they would be used despite not showing a signature profile for the selected feature. Furthermore, synthetically adding gene set signatures one at a time may not correctly represent biological signatures that can coexist together. As a result, our proposed approach aims to be as biologically consistent as possible. Our proposed solution differs from the original MCS algorithm in two aspects: feature presence consideration and data sampling. First, original MCS solution uses the entire dataset to calculate contribution scores, however, given the sparsity of gene pair features there will be many cases where a feature will yield the same performance when it is in and out of the coalition (i.e., null feature, yields a Shapley value of 0). By only considering the gene pairs that have a value for the given feature (i.e., not null), we significantly reduce the computational cost in SL/GCE models built on molecular signature data.

Second, our implementation effectively uses data sampling by considering the feature space in the dataset rather than random sampling. Due to each gene pair being unique and certain SL/GCE datasets having thousands of gene pair combinations, running multiple sampling iterations in a very high dimensionality setting is still not feasible. Instead, by calculating the Shapley values directly from gene pairs that contain the select features and the scenarios that they don’t, the coalition of the features stay true to the gene pair data. Each gene pair ideally has their own unique feature space, thereby effectively using data sampling in the process and does not rely on additional sampling iterations as done in traditional approaches. This step, like before, further cuts down the computational costs, and retains the biologically accurate Shapley value calculations in SL/GCE models.

## 3 Detailed data preparation for transfer learning

#### Selected gene-combination double-knockout (CDKO) experiments and data are presented in Table 1. Table is also presented in the main text of the paper.

#### 3.1 Baseline Non-TL models

Under this selection, every study’s cell line data were separately trained for both SL and GCE tasks. For SL, select nine studies and their number of cell lines are trained: Han et al. 2017 – 1, Horlbeck et al. 2018 – 2, Ito et al. 2021 – 11, Parrish et al. 2021 – 2, Shen et al. 2017 – 2, Tang et al. 2021 – 1, Thompson et al. 2021 – 2, Wong et al. 2016 – 1, and Zhao et al. 2018 – 2. For GCE, the same datasets as SL were used except Han et al. 2017 due to no sgRNA counts. As a result, 24 SL and 23 GCE models were trained for a total of 240 SL and 230 GCE scenarios over 10 computational runs.

*Example*: A model is trained on Horlbeck et al. 2018’s K562 cell line data. No TL or any special considerations are applied, as a result this is a regular SL prediction model.

**Table 1.** Selected gene-combination double-knockout (CDKO) experiments from the Synthetic Lethality Knowledge Base (SLKB)

| **CDKO studies** | **Numbers of genes**  **and gene pairs*** | **Cell lines** | **Gene selection mechanisms** |
| --- | --- | --- | --- |
| Wong et al. 2016 (1) | 48 genes,  1,128 gene pairs | OVCAR8 | Epigenetics regulation in ovarian cancer |
| Han et al. 2017 (2) | 203 genes,  20,387 gene pairs | K562 | **Drug targets** |
| Shen et al. 2017 (3) | 73 genes,  2,628 gene pairs | HeLa, A549 | **Drug targets** include tumor suppressors |
| Horlbeck et al. 2018 (4) | 425 genes,  89,430 gene pairs | Jurkat, K562 | **Non-essential genes** |
| Zhao et al. 2018 (5) | 50 genes,  1,225 gene pairs | A549, HeLa | **Metabolic pathways** |
| Ito et al. 2021 (6) | 3,192 genes,  4,933 paralog pairs | A549, GI-1, HS 936.T, HS 944.T, HSC-5, IPC-298, MEL-202, MEL-JUSO, MeWo, PA-TU-8988S, PK-1 | **Paralog families** |
| Parrish et al. 2021 (7) | 2,000 genes,  1,000 gene pairs | HeLa, PC-9 | **Paralog families** |
| Thompson et al. 2021 (8) | 1,566 genes,  1,125 gene pairs | A-375, MeWo | **Paralog families** |
| Tang et al. 2022 (9) | 50 genes,  1,225 gene pairs | 22Rv1 | **Cell death pathways and drug targets** |

#### 3.2 Pretraining models for TL applications

To be used in TL applications only, new models were trained. ‘Same genes different cells’ and ‘same cell different genes’ TL applications were carried out within studies and across studies respectively, using the same dataset as non-TL models. To allow TL in those scenarios, we train new models using the same datasets but with the entire datasets, creating 24 SL and 23 GCE source models for a total of 240 SL and 230 GCE source models over 10 different runs.

*Example*: The source model is trained on Horlbeck et al. 2018’s Jurkat cell line data with the entire dataset. This model will later be used in TL tasks to help improve predictions in Horlbeck et al. 2018’s K562 cell line data in same genes and different cells TL application. Another source model is trained on entirety of Han et al. 2017’s K562 cell line data. This model will also be used in TL to target dataset Horlbeck et al. 2018’s K562 cell line data in same cell and different genes TL application.

For similar mechanism TL application, groups of datasets were combined based on their gene selection mechanisms to act as an umbrella term to curate studies under (**Table 1).** There are two groups: paralog families (Ito et al. 2021, Parrish et al. 2021, and Thompson et al. 2021), and drug targets (Han et al. 2017, Shen et al. 2017). For every group, the TL target study was held out while the remaining studies were combined to train the model that will be used for TL. Then, the trained model will be used for TL in all of target study’s cell lines one by one. As a result, paralog families group creates 3 SL and GCE source models and drug targets group creates 2 SL and no GCE source models (Han et al. 2017 does not have GCE data), totaling 5 SL and 3 GCE source models. Over 10 computational runs, we obtain 50 SL and 30 GCE source models.

*Example*: To be used for Thompson et al. 2021 data, all cell line data from Ito et al. 2021 and Parrish et al. 2021 are used to train a source model. This model will then be used in TL applications for predicting Thompson et al. 2021’s A-375 cell line data in similar mechanism application.

For big data to small data TL application, models are trained on all SLKB data with the target study’s cell line data held out, creating 24 SL and 23 GCE source models. To sum up, a total of 57 SL and 55 GCE source models were trained specifically to use for TL in one computational run, and 570 SL and 550 GCE source models over 10 computational runs.

*Example*: The source model is trained with entire SLKB data except Horlbeck et al. 2018’s K562 cells. Then, this model is used in TL applications for Horlbeck et al. 2018’s K562 cell line data under big data to small data application.

#### 3.3 Same gene and different cell TL application

Here, TL is applied within studies that target multiple cell lines, this encompasses Horlbeck et al. 2018 (2 cells), Ito et al. 2021 (11 cells), Parrish et al. 2021 (2 cells), Shen et al. 2017 (2 cells), and Zhao et al. 2018 (2 cells). Within each study, cells are applied TL from one cell to another in combination. As a result, many combinations are present (e.g., Horlbeck et al. 2018 has 2, Ito et al. 2021 has 110, Parrish et al. 2021 has 2). Including ensemble model, there were five total TL methods applied for each TL instance. This yields 120 x 5 = 600 SL and GCE TL instances, with most originating from Ito et al. 2021. Across 10 computational runs, this creates 6,000 SL and GCE TL instances.

*Example*: Horlbeck et al. 2018 has two cells: Jurkat and K562. The source model was trained on entire data of Horlbeck et al. 2018’s Jurkat cell line. The model is used via TL to train on Horlbeck et al. 2018’s K562 cell line dataset. Similarly, the source model that was trained on Horlbeck et al. 2018’s entire K562 cell line data will be used to help predict Jurkat cell line dataset.

#### 3.4 Same cell and different gene TL application

For this scenario, TL is applied on cell lines that are targeted by multiple studies. There are four cell lines that were present across multiple studies: A549 (Shen et al. 2017, Zhao et al. 2018, and Ito et al. 2021), HeLa (Shen et al. 2017, Zhao et al. 2018, and Parrish et al. 2021), K562 (Han et al. 2017 and Horlbeck et al. 2018), and MeWo (Horlbeck et al. 2018 and Thompson et al. 2021). Each cell line produces 6, 6, 2, and 2 combinations of studies respectively. Thus, including the five TL methods, there are 16 x 5 = 80 SL and 14 x 5 = 70 GCE TL instances in one computational run, yielding 800 SL and 700 GCE TL instances across all 10 computational runs.

*Example*: K562 cells were screened in two studies: Han et al. 2017 and Horlbeck et al. 2018. The source model was trained on all of Han et al. 2017’s K562 cell line data. Then, this model is used via TL to train on Horlbeck et al. 2018’s K562 cell line data. Similarly, a source model trained on Horlbeck et al. 2018’s all K562 cell data can be used in TL tasks to help improve predictions on Han et al. 2017’s K562 cell line data.

#### 3.5 Same mechanism TL application

In this application, each study’s cell line is applied TL only once using its mechanistic group’s respective model. As each study is under a single gene selection mechanism, each study’s cell data is applied TL only once. As a result, there are 18 x 5 = 90 SL and 15 x 5 = 75 GCE TL instances. Applied on 10 computational runs, this results in 900 SL and 750 GCE TL instances.

*Example*: Thompson et al. 2021 has mainly targeted paralog genes in its study design, therefore it has been categorized as a paralog family targeting study. In this scenario, source model is trained with entire data from all studies targeting paralog genes except Thompson et al. 2021; consisting of only Ito et al. 2021’s and Parrish et al. 2021’s all cell data. Then, this model is used in TL tasks for Thompson et al. 2018’s A-375 cells. This is repeated for Thompson et al. 2018’s MeWo cells as well.

#### 3.6 Big data to small data TL application

Similar to similar mechanism TL application, TL is applied only once to each dataset using the model trained on entire SLKB data with the query cell line data held out. As a result, the number of TL instances are identical to the same mechanism application; 120 SL and 115 GCE TL instances. Over 10 computational runs, this application yields 1,200 SL and 1,150 GCE TL scenarios.

*Example*: The source model is trained on entire SLKB database, except Horlbeck et al. 2018’s K562 cell line data. Then, this model is used in TL applications for target dataset Horlbeck et al. 2018’s K562 cell line data, the data that was previously held out.

## 4 Assumptions in transfer learning for synthetic lethal experiments

Each CDKO SL study is unique; having a specific study design (e.g., targeting certain genes and cells), utilizing their own *in silico* SL identification methods, and screening SL experiments with custom CRISPR-Cas9 backbone and plasmids. Considering experimental conditions and systems biology, each data source is highly different from each other. Therefore, several assumptions must be made in order to employ TL strategies between various studies:

1) All CDKO screens target genes and gene pairs that are expressed in the cell line. Probable missense mutations across cell lines ideally do not contribute towards essentiality, i.e., targeting genes in different cells yield results respective to context specificity of the cell and not a probable missense mutation.
2) All CDKO sgRNAs target their intended genes with maximal on-target effects and minimal off-target effects.
3) All negative control sgRNAs, and their targeted regions, behave the same; have no impact on cell viability and have no function outside of measuring cell growth without perturbation.
4) All CDKO experiments share the same efficacy and efficiency.
5) Study reported SL scores accurately portray cell viability in CDKO screens. The impacts of different SL scores are marginal.

These assumptions are necessary in order to carry out transfer learning across applications outlined in **Supplementary Figure 1.** It is important to note that while these assumptions make each study more comparable, all studies remain unique through their targeted genes and cells. These differences are then used to design TL applications appropriate for each dataset.

## 5. SL performance metrics

Accuracy: $\frac{TP+TN}{TP+FP+TN+FN}$

Precision: $\frac{TP}{TP+FP}$

Recall: $\frac{TP}{TP+TN}$

AUC-ROC: $\int_{0}^{1} TPR(FPR) d(FPR)$s.t. *TPR(FPR)* returns true positive rate at given false positive rate.

AUPR: $\int_{0}^{1} Precision\left( Recall \right) d(Recall)$s.t. *Precision(Recall)* returns precision at given recall.

AUC-ROC @ k: $\int_{0}^{1} TPR(FPR) d(FPR)$s.t. *TPR(FPR)* returns true positive rate at given false positive rate and *FPR* < k

Precision @ k: $\frac{TP[k]}{TP[k]+TN[k]}$ s.t. TP[k] and TN[k] return metrics for the top k% prediction

TP: True positive, TN: True negative, FP: False positive, FN: False negative, TPR: True positive rate, FPR: False positive rate.

## 6. GCE performance metrics

Spearman rank correlation coefficient: $1- \frac{6\times\sum_{i=1}^{n} d_{i}^{2}}{n\times(n^{2}-1)}$, $d_{i}$: Distance between ranks of variable $i$ between two groups. n: Sample size.

Pearson correlation coefficient: $\frac{\sum_{i=1}^{n} (x_{i}-\bar{x})(y_{i}-\bar{y})}{{\int\sum_{i=1}^{n} \left( x_{i}-\bar{x} \right)^{2}\sum_{i=1}^{n} \left( y_{i}-\bar{y} \right)}^{2}}$, $x_{i}$ and $y_{i}$ indicate regression values for variable $i$, and $\bar{x}$ and $\bar{y}$ indicate the average of the variables within groups.

Correlation coefficient p-value: $\sqrt{\frac{r^{2}\times(n-2)}{1-r^{2}}}$, $r$: Correlation coefficient. $n:$Sample size

**Reference**

1.	Wong AS, Choi GC, Cui CH, Pregernig G, Milani P, Adam M, et al. Multiplexed barcoded CRISPR-Cas9 screening enabled by CombiGEM. Proc Natl Acad Sci U S A. 2016;113(9):2544–9.

2.	Han K, Jeng EE, Hess GT, Morgens DW, Li A, Bassik MC. Synergistic drug combinations for cancer identified in a CRISPR screen for pairwise genetic interactions. Nat Biotechnol. 2017;35(5):463–74.

3.	Shen JP, Zhao D, Sasik R, Luebeck J, Birmingham A, Bojorquez-Gomez A, et al. Combinatorial CRISPR-Cas9 screens for de novo mapping of genetic interactions. Nat Methods. 2017;14(6):573–6.

4.	Horlbeck MA, Xu A, Wang M, Bennett NK, Park CY, Bogdanoff D, et al. Mapping the Genetic Landscape of Human Cells. Cell. 2018;174(4):953–67 e22.

5.	Zhao D, Badur MG, Luebeck J, Magana JH, Birmingham A, Sasik R, et al. Combinatorial CRISPR-Cas9 Metabolic Screens Reveal Critical Redox Control Points Dependent on the KEAP1-NRF2 Regulatory Axis. Mol Cell. 2018;69(4):699–708 e7.

6.	Ito T, Young MJ, Li R, Jain S, Wernitznig A, Krill-Burger JM, et al. Paralog knockout profiling identifies DUSP4 and DUSP6 as a digenic dependence in MAPK pathway-driven cancers. Nat Genet. 2021;53(12):1664–72.

7.	Parrish PCR, Thomas JD, Gabel AM, Kamlapurkar S, Bradley RK, Berger AH. Discovery of synthetic lethal and tumor suppressor paralog pairs in the human genome. Cell Rep. 2021;36(9):109597.

8.	Thompson NA, Ranzani M, van der Weyden L, Iyer V, Offord V, Droop A, et al. Combinatorial CRISPR screen identifies fitness effects of gene paralogues. Nat Commun. 2021;12(1):1302.

9.	Tang S, Wu X, Liu J, Zhang Q, Wang X, Shao S, et al. Generation of dual-gRNA library for combinatorial CRISPR screening of synthetic lethal gene pairs. STAR Protoc. 2022;3(3):101556.

10.	Paszke A, Gross S, Massa F, Lerer A, Bradbury J, Chanan G, et al. PyTorch: An Imperative Style, High-Performance Deep Learning Library. Adv Neur In. 2019;32.

11.	Kingma DP. Adam: A method for stochastic optimization. arXiv preprint arXiv:14126980. 2014.

12.	Rozemberczki B, Watson L, Bayer P, Yang HT, Kiss O, Nilsson S, et al. The Shapley Value in Machine Learning. Proceedings of the Thirty-First International Joint Conference on Artificial Intelligence, Ijcai 2022. 2022:5572–9.

13.	Shrikumar A, Greenside P, Kundaje A. Learning Important Features Through Propagating Activation Differences. Pr Mach Learn Res. 2017;70.

14.	Chen H, Lundberg SM, Lee SI. Explaining a series of models by propagating Shapley values. Nat Commun. 2022;13(1).

15.	Hastings WK. Monte-Carlo Sampling Methods Using Markov Chains and Their Applications. Biometrika. 1970;57(1):97–&.

16.	Mitchell R, Cooper J, Frank E, Holmes G. Sampling Permutations for Shapley Value Estimation. J Mach Learn Res. 2022;23:1–46.
